# GPR65 sensing tumor-derived lactate induces HMGB1 release from TAM via the cAMP/PKA/CREB pathway to promote glioma progression

**DOI:** 10.1186/s13046-024-03025-8

**Published:** 2024-04-04

**Authors:** Chaolong Yan, Zijiang Yang, Pin Chen, Yuyang Yeh, Chongjing Sun, Tao Xie, Wei Huang, Xiaobiao Zhang

**Affiliations:** 1 Department of Neurosurgery, Zhongshan Hospital, Fudan University, Shanghai, China; 2 State Key Laboratory of Neuroscience, Institute of Neuroscience, Center for Excellence in Brain Science and Intelligence Technology, Chinese Academy of Sciences, Shanghai, China

**Keywords:** Glioma, Lactate, Tumor-associated macrophage (TAM), GPR65, HMGB1

## Abstract

**Background:**

Lactate has emerged as a critical regulator within the tumor microenvironment, including glioma. However, the precise mechanisms underlying how lactate influences the communication between tumor cells and tumor-associated macrophages (TAMs), the most abundant immune cells in glioma, remain poorly understood. This study aims to elucidate the impact of tumor-derived lactate on TAMs and investigate the regulatory pathways governing TAM-mediated tumor-promotion in glioma.

**Methods:**

Bioinformatic analysis was conducted using datasets from TCGA and CGGA. Single-cell RNA-seq datasets were analyzed by using UCSC Cell Browser and Single Cell Portal. Cell proliferation and mobility were evaluated through CCK8, colony formation, wound healing, and transwell assays. Western blot and immunofluorescence staining were applied to assess protein expression and cell distribution. RT-PCR and ELISA were employed to identify the potential secretory factors. Mechanistic pathways were explored by western blotting, ELISA, shRNA knockdown, and specific inhibitors and activators. The effects of pathway blockades were further assessed using subcutaneous and intracranial xenograft tumor models in vivo.

**Results:**

Elevated expressions of LDHA and MCT1 were observed in glioma and exhibited a positive correlation with M2-type TAM infiltration. Lactate derived from glioma cells induced TAMs towards M2-subtype polarization, subsequently promoting glioma cells proliferation, migration, invasion, and mesenchymal transition. GPR65, highly expressed on TAMs, sensed lactate-stimulation in the TME, fueling glioma cells malignant progression through the secretion of HMGB1. GPR65 on TAMs triggered HMGB1 release in response to lactate stimulation via the cAMP/PKA/CREB signaling pathway. Disrupting this feedback loop by GPR65-knockdown or HMGB1 inhibition mitigated glioma progression in vivo.

**Conclusion:**

These findings unveil the intricate interplay between TAMs and tumor cells mediated by lactate and HMGB1, driving tumor progression in glioma. GPR65, selectively highly expressed on TAMs in glioma, sensed lactate stimulation and fostered HMGB1 secretion via the cAMP/PKA/CREB signaling pathway. Blocking this feedback loop presents a promising therapeutic strategy for GBM.

**Supplementary Information:**

The online version contains supplementary material available at 10.1186/s13046-024-03025-8.

## Introduction

Glioma, is the most common and aggressive primary intracranial tumors, accounting for over 40% of primary intracranial tumors [1]. According to the World Health Organization (WHO) classification system, glioblastoma (GBM) is categorized as grade IV glioma, representing the highest degree of malignancy [2]. Despite the availability of aggressive treatments such as surgical resection, chemotherapy, and radiation for GBM patients, the median survival rate remains below 15 months, often marked by rapid recurrence and progression within one-year post-operation [3]. Therefore, exploring the potential pathogenic mechanisms involved in tumor progression is of great significance for the development of novel therapeutic strategies and, ultimately, to improve the prognosis for glioma, even GBM patients.

Based on the molecular characteristic of tumors and the correlation with patients’ prognosis, glioma has been classified into four distinct subtypes: proneural (PN), neural (NL), classical (CL) and mesenchymal (MES) [4]. The MES subtype is the most aggressive with the worst prognosis and can transform from PN subtype during tumor progression [5, 6]. To date, extensive studies have focused on the intrinsic factors involved in cancer development and progression. However, how the mechanisms by which extrinsic signals drive tumor progression remain largely elusive. The tumor microenvironment (TME) encompasses a diverse array of cellular components, including immunocytes, vascular endothelial cells and extracellular matrix, all of which play an important role in regulating the malignant progression of tumor cells [7, 8]. Among these components, macrophages are the most abundant immune cells in the glioma TME, accounting for more than 30%, and in some cases, up to 70% of infiltrating cells [9–11]. Macrophages can be polarized into two states: M1-subtype (classically activation) and M2-subtype (alternative activation), induced by various factors. M1-macrophages typically produce pro-inflammatory factors and exert anti-tumor effects, whereas M2-macrophages predominantly secret anti-inflammatory cytokines, promoting tumor progression [12]. Typically, macrophages infiltrated in tumors, named tumor-associated macrophages (TAM), shows M2-polarization characteristics and promote tumor malignant progression by secreting a variety of cytokines (*e.g.*, TGFB1 [13] and HMGB1 [14]) and chemokines (*e.g.*, CCL17 [15] and CXCL7 [16]). However, the complex crosstalk between glioma cells and TAMs, explaining how glioma cells induce M2-polarized macrophages and how M2-TAMs facilitate glioma progression through factor secretion, remains unclear.

Recently, studies have highlighted the impact of elevated lactate in the TME on modulating the signaling function and regulating the M2-polarization of macrophages, thereby facilitating the malignant progression in tumors [14, 15, 17–19]. Under physiological conditions, cellular energy metabolism primarily depends on the oxidative phosphorylation in normoxic conditions and glycolysis in hypoxic conditions. However, in most solid tumors, aerobic glycolysis is recognized as the primary energy source for rapidly proliferating cancer cells, known as Warburg effect. This metabolic shift consistently contributes to elevated levels of lactate and an acidified TME [20]. The accumulation of lactate in the TME is a significant metabolic stimulus in regulating cancer progression. Studies have reported that lactate dehydrogenase A (LDHA)-mediated lactate production increases in glioma, especially in IDH wild-type glioma and monocarboxylate transporter 1 (MCT1) plays a prominent role in transmembrane transport of lactate [21–25]. Moreover, elevated serum lactate was reported to be correlated with glioma grade, a higher Ki-67 index and larger tumor volumes, indicating a poorer prognosis [26–28]. However, the precise mechanism through which lactate in the TME influences signaling function of TAMs and consequently promotes the malignant progression of glioma remains elusive.

In this study, we found that lactate metabolism was upregulated in glioma and its high levels in TME stimulated TAMs towards the tumor-promoting M2-subtype. Furthermore, a proton-sensing G-protein coupled receptors (GPRs), GPR65 was identified as the main receptor sensing lactate-signal on TAMs, mediating the protumor effects of TAMs in glioma. Mechanistically, the lactate-signal activated GPR65 on TAMs, triggering the secretion of HMGB1 through the cAMP/PKA/CREB signaling pathway, ultimately facilitating glioma progression. In summary, our findings highlight the dynamic crosstalk between TAMs and tumor cells mediated by lactate and HMGB1, which plays a pivotal role in promoting glioma progression. Blocking this feedback loop by targeting GPR65 or HMGB1 represents an attractive therapeutic option for glioma.

## Methods

### Reagents

Compounds and reagents include: lactate (#L6402, Sigma-Aldrich, USA), phorbol 12-myristate 13-acetate (PMA, #S7791, Selleck Chemicals, USA), LDHA inhibitor, sodium oxamate (SO, #6871, Selleck Chemicals, USA), MCT inhibitor, a-cyano-4-hydroxycinnamate (CHC, #S8612, Selleck Chemicals, USA), HMGB1 inhibitor, glycyrrhizin (#HY-N0184, MedChemExpress, USA), adenylate cyclase inhibitor, SQ22536 (#HY-100396, MedChemExpress, USA), PKA inhibitor, H89 (#HY-15979, MedChemExpress, USA), Forskolin (#HY-15371, MedChemExpress, USA), rHMGB1 (#ab167718, Abcam, USA).

### Human tissue samples

The study was conducted in accordance with the Declaration of Helsinki and approved by the Ethical Committee of Zhongshan Hospital, Fudan University. All patients included in the study had written informed consent for the use of clinical data and specimens. Human glioma tissue and non-tumor samples were collected between January 2021 and August 2023 from the Department of Neurosurgery, Zhongshan Hospital of Fudan University. Non-tumor samples were obtained from the corticostomy while exposing the deep tumor during surgery, collecting as the negative control group. Histopathological diagnosis was performed by at least two neuropathologists based on the World Health Organization (WHO) classification.

### Bioinformatic analysis

All expression profiling data of mRNA analyzed in the study was download from the public website: The Cancer Genome Atlas (TCGA, http://cancergenome.nih.gov) and The Chinese Glioma Genome Atlas (CGGA, http://www.cgga.org.cn). The data was analyzed by the web-based tools GEPIA (http://gepia.cancerpku.cn/) and Gliovis websites (http://gliovis.bioinfo.cnio.es/) [29]. The correlated analysis was performed by GraphPad Prism (version 8) by using datasets from TCGA and CGGA. The online single-cell RNA-seq dataset UCSC Cell Browser (https://gbm.cells.ucsc.edu) [30, 31] and Single Cell Portal (https://singlecell.broadinstitute.org, GSE131928) [11] were used to visualize the genes expression of cell type in GBM.

### Cell culture and transfection

Cell lines U87-MG (U87), U251 and THP1 were purchased from Cell Bank of the Chinese Academy of Sciences (Shanghai, China). All cells were maintained in a controlled environment with 5% CO2 at 37℃. U87 and U251 cell lines were cultured in Dulbecco’s modified Eagle’s medium (DMEM, Gibco, Waltham, USA), supplemented with 10% fetal bovine serum (FBS, Gibco) and 1% antibiotic mixture (Gibco). Additionally, three primary human GBM cell lines were established in our laboratory, derived from surgical samples of three GBM patients. The tissue specimens were dissected into 1 × 1 × 1 mm ^3^ pieces and digested with 0.25% trypsin at a volume 2–3 times that of the tissue, maintained at 37 ℃ for 15–20 min. The resulting cell suspension was collected, filtered, and centrifuged at 1000 rpm for 5 min. The pelleted cells were resuspended in complete culture medium and cultured in DMEM supplemented with 10% FBS. The monolayer cells were harvested as primary GBM cells by detaching them with 0.25% trypsin.

THP1 cells were cultured in RPMI-1640 medium (Gibco) supplemented with 10% FBS and 1% antibiotic mixture. To generate THP1-derived M0 macrophages (M0), THP1 cells were treated with 10 ng/ml PMA (Sigma) for 24 h. And then M0 macrophages were stimulated with lactate or various conditioned mediums. To mimic the formation of TAMs, M0 macrophages were co-cultured with glioma cells (U87, U251 or GBM3) using a 6-well transwell co-culture system (0.4 μm pore size, Corning, USA). After 48 h, the co-cultured macrophages were collected to obtain TAMs. For the knockdown of GPR65, TAMs were transfected with lentiviruses carrying shRNA-GPR65 (shGPR65) or shRNA-NC (shNC), which were acquired from Genomeditech Co., Ltd. (Shanghai, China). The efficiency of transfection was verified by qPCR and western blot analysis.

### Conditioned medium (CM) collection

To prepare the conditioned medium, the cells were cultured with serum-free medium following corresponding treatments. After a 48-h incubation period, the cultured supernatant was harvested for subsequent centrifugation and filtration with 0.2 μm filters, which was defined as conditioned medium (CM). For U87, U251 and GBM3 cells, three different seeding concentrations were used as 2.5 × 10^5^, 5 × 10^5^, 1 × 10^6^ cells/well, and the corresponding supernatants were collected after 48 h, designated as CM1, CM2, CM3, respectively. Primary human glioblastoma cells were seeded in 6-well plates at 1 × 10^6^ cells/well, and the culture supernatants were collected after 48 h, denoted as GBM-derived CM.

### Cell Counting Kit-8 (CCK-8) assay

The Cell Counting Kit-8 (CCK-8) assay was employed to assess cell viability following various treatments, in accordance with the guidelines provided by the manufacturer (Beyotime, Shanghai, China). Tumor cells were seeded at a density of 4 × 10^3^ cells per well in 96-well plates. Subsequently, 10 μL of the CCK-8 reagent was added to each well and incubated at 37 ℃ for 1.5 h. The absorbance at 450 nm (OD_450nm_) of each well was measured at 24, 48, 72 and 96 h using a Microplate Reader (Bio-Rad, Hercules, USA). The results were representative of three independent experiments, each of which consisted of three replicates.

### Colony formation assay

For the colony formation assay, cells were digested and resuspension into single cells, and 1 × 10^3^ cells/well were seeded into 6-well plate. The cells were cultured in complete medium or corresponding CMs with the culture medium refreshed every three days. After incubated for 14 days, cells were rinsed with PBS three times, fixed with 4% paraformaldehyde for 20 min and stained with 0.1% crystal violet for 20 min. After an additional washing with PBS three times, the representative colonies were visualized and counted.

### Migration and invasion evaluation

Cell migration and invasion capabilities were measured by wound healing and transwell assays.

Wound healing assay was performed to evaluate cell migration capacities. Tumor cells following pre-treatments were initially seeded into six-well plates and incubated at 37℃ until they reach over 90% confluence. Subsequently, a defined scratched wound was meticulously created by gently scraping the cell monolayer with 200 μL pipette tips and the detached cells were removed by washing with PBS. The cells were then allowed to culture for 24 h for further study. Images were captured at both 0-h and 24-h under a light microscope (Olympus, Japan). The pictures were analyzed using Image J software, and the wound healing rate was calculated as the formula [(wound area at 0 h)—(wound area at 24 h)] / (wound area at 0 h) × 100%.

The transwell assay was conducted to assess cell migration (without Matrigel) and invasion (with Matrigel) capacities by using 24-well transwell chambers with 8 μm pore size (Corning, USA). For the migration assay, 4 × 10^4^ cells following pre-treatments were suspended in 200 μL serum-free DMEM and seed into the upper chamber, while 700 μL complete medium with 10% FBS was added into the lower chamber. After a 24-h incubation, the non-invading cells were carefully removed from the upper surface of the upper chamber using a cotton tip. The invading cells adhering to the lower surface were fixed with 4% paraformaldehyde for 20 min and stained with 0.1% crystal violet for 20 min. Images were captured from five random fields of each well under an inverted microscope (Olympus). The results were calculated from three replicates of each experiment. For the invasion assay, the transwell membranes were pre-coated with Matrigel (BD Biosciences, USA) before seeding cells. The remaining steps were the same as described above.

### Western blot

The total protein from the samples was extracted using RIPA lysis buffer (Beyotime, Shanghai, China), which contained protease inhibitors (Beyotime). The protein concentration was determined with a BCA Protein Assay Reagent Kit (Beyotime). Equal amounts of proteins were separated on a 4–15% SDS-PAGE gel and subsequently transferred onto 0.45 μm PVDF membranes (Merck Millipore, Billerica, USA). After blocking with 5% skim milk for 1 h at room temperature, the membranes were incubated with the primary antibodies overnight at 4℃. Afterward, the membranes were washed with TBST and then incubated with HRP-combined secondary antibodies for 1 h at room temperature. The list of antibodies used is provided in Table S1. The protein bands were visualized using an enhanced chemiluminescence kit (Thermo Fisher Scientific, Waltham, MA), and the bands densities were analyzed with ImageJ software (NIH, United States).

### Real-time quantitative polymerase chain reactions

Total RNA was extracted from the samples using Trizol reagent (Thermo Fisher Scientific) following the manufacturer's instructions. Subsequently, cDNA was synthesized using a reverse transcription (RT) reagent kit (Vazyme, China). Quantitative PCR was performed on the PowerUp SYBR Green Master Mix kit (Vazyme). The primer sequences used are provided in Table S2, and GAPDH was used as internal reference for mRNA quantification. The relative expression levels of the target mRNAs were calculated by the 2^−ΔΔCt^ method.

### Lactate, HMGB1 and cAMP measurement

Concentrations of lactate and HMGB1 from cell culture supernatants, and intracellular cAMP were measured by using separate ELISA Kits, Lactate (ab65330, Abcam, USA), HMGB1 (PH406, Beyotime Biotechnology, China) and cAMP (ab234585, Abcam) according to the manufacturer’s instructions, respectively. The prepared reaction mixture was incubated at room temperature for 30 min protected from light and measured output on microplate reader. Results were calculated as the protocol indicated.

### Immunofluorescence staining

For immunofluorescence staining of cells, cells were seeded in glass slides. After various stimulating treatments, the cells were washed with PBS and fixed with pre-cooled methanol for 20 min. After washing with PBS three times, the cells were incubated with the primary antibodies with 0.1% Triton X-100 and 1% BSA at 4℃ overnight. The secondary antibodies along with DAPI were applied at room temperature for 120 min. The list of antibodies is provided in Table S1. Images were visualized and photographed using a fluorescence microscope (Olympus).

For immunofluorescence staining of human tissue samples, the tissues were fixed in 4% paraformaldehyde for 24 h and then subjected to a sucrose gradient for dehydration. Subsequently, specimens were embedded in OCT and sectioned into 20 μm slices. After washing with PBS three times, the slices were incubated with the primary antibodies with 0.5% Triton X-100 and 1% BSA at 4℃ overnight. The secondary antibodies, along with DAPI, were applied at room temperature for 120 min. The list of antibodies is provided in Table S1. Images were visualized and photographed using a fluorescence microscope (Olympus).

### In vivo xenograft tumor models

All animal experiments were performed in strict adherence to the ethical principles and guideline of the National Institutes of Health for the care and use of animals, and approved by the Committee on Animal Research of Zhongshan Hospital, Fudan University. Six to eight-week-old BALB/c Nude mice were purchased from Vital River Laboratory Animal Technology Co. (Beijing, China) and were housed in a specific pathogen-free (SPF) animal facility.

For the subcutaneous xenograft tumor model, 100 μL cell suspensions of U87 cells and TAMs were subcutaneously injected on the right flank of BABL/c-nude mice (*n* = 4/group). Mice were randomly separated into four groups: I: 1 × 10^6^ U87 cells, II: 1 × 10^6^ U87 + 1 × 10^6^ shNC-TAM cells, III: 1 × 10^6^ U87 + 1 × 10^6^ shGPR65-TAM cells, IV: 1 × 10^6^ U87 + 1 × 10^6^ shNC-TAM cells + glycyrrhizin. Glycyrrhizin was injected around the xenograft tumor at 3-day intervals with 1 nM/kg starting from 28 days post-modeled. Tumor sizes were measured once a week and calculated as: volume = length × width ^2^/2. Six weeks after of injection, the tumors were collected, weighed, and stored for further studies.

For the orthotopic glioma model, it was established by intracranial implantation of 5 μL cell suspensions of U87 cells and TAMs. Mice were randomly separated into four groups: I: 2 × 10^5^ U87 cells, II: 2 × 10^5^ U87 + 2 × 10^5^ shNC-TAM cells, III: 2 × 10^5^ U87 + 2 × 10^5^ shGPR65-TAM cells, IV: 2 × 10^5^ U87 + 2 × 10^5^ shNC-TAM cells + glycyrrhizin. Glycyrrhizin was intracerebroventricular injected at 3-day intervals with 1 nM/kg starting from 14 days post-modeling. In brief, the mice were anesthetized and secured in a stereotaxic instrument (RWD Life Science, China). A burr hole was made on the skull using a sterile drill at 2 mm anterior the bregma, 2 mm on the right of the midline. Cell suspensions were injected to the depth of 2 mm below to the surface of the skull. Following the implantation, the incision was sutured with silk sutures. The mice were closely monitored until they exhibited moribund signs and the survival days were recorded. Then the mice were anesthetized for specimen collection for further study.

### Statistical analysis

All experiments were performed in triplicate. Data was analyzed by GraphPad Prism (version 8.0, USA) and presented as mean ± SD. Student’s t-test was adopted for the analysis between two groups. *p* < 0.05 indicated a statistically significant difference.

## Results

### Lactate metabolism was increased in glioma and positively correlated with M2-type TAM infiltration

Due to the Warburg effect, most solid tumors exhibit an upregulation in lactate metabolism, which leads to the acidification of the tumor microenvironment (TME). In glioma, lactate production is primarily mediated by lactate dehydrogenase A (LDHA), while the efflux is mainly facilitated by monocarboxylate transporter 1 (MCT1) [21–25].

**Fig. 1:**
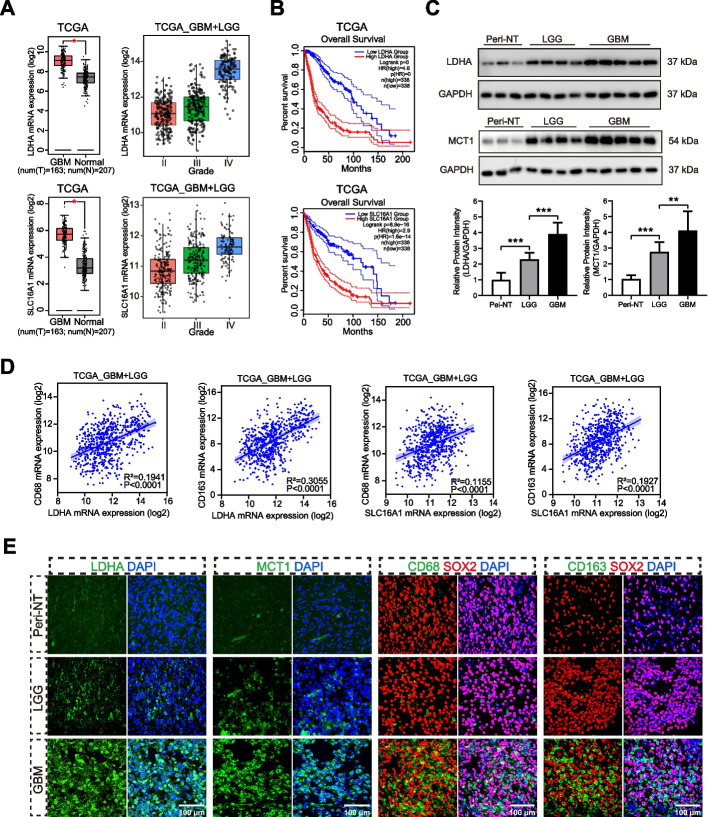
Lactate metabolism was increased in glioma and positively correlated with M2-type TAM infiltration. **A** Different mRNA expression levels of LDHA or SLC16A1, between *GBM* and *Normal* group, or among grade of gliomas in TCGA dataset. **B** Kaplan–Meier survival curves revealed the correlation between LDHA or SLC16A1 mRNA expression and survival of glioma patients in the TCGA dataset. **C** Western blot analysis of LDHA and MCT1 protein expression in clinical samples. **D** Correlation analysis between the expressions of LDHA or SLC16A1 and CD68 or CD163, respectively, using bulk RNA-seq data from the TCGA dataset. **E** Representative pictures for immunofluorescence staining of clinical tissues for LDHA, MCT1, CD68 and CD163. Cell nuclei were counterstained with DAPI. Data are presented as the Mean ± SEM. *p < 0.05, **p < 0.01, and ***p < 0.001

To evaluate the levels of lactate metabolism in glioma, we analyzed the mRNA levels of LDHA and MCT1 (referred as SLC16A1 at the mRNA level) using bulk RNA-seq data from the TCGA and CGGA datasets. Our analysis revealed that the expression of LDHA in glioma was significantly elevated compared to normal tissues (Figs. 1A & S1A). Furthermore, the expression levels increased in tandem with the higher WHO grade of glioma. Kaplan–Meier analysis results revealed that high LDHA expression correlated with poor overall survival (OS) among glioma patients in both TCGA and CGGA datasets (Figs. 1B & S1A). Similar results were observed for the analysis of SLC16A1 (Figs. 1A & S1A). Subsequently, we assessed the protein levels of LDHA and MCT1 using clinical samples through western blot analysis. Consistent with the bioinformatics analysis, the results indicated increased protein expressions of LDHA and MCT1 in gliomas compared to peri-tumorous normal tissues (Peri-NT), with notably higher levels in glioblastomas (GBM) compared to low-grade gliomas (LGG) (Fig. 1C).

To investigate the relationship between lactate levels and macrophage infiltration, we performed correlation analysis involving lactate metabolism markers LDHA (for lactate production) and SLC16A1 (for lactate efflux), along with macrophages markers CD68 (for total macrophage infiltration) and CD163 (for M2-macrophage infiltration) using bulk RNA-seq data from the TCGA and CGGA datasets. The results demonstrated a positive correlation between the expressions of LDHA or SLC16A1 and CD68 or CD163, respectively (Figs. 1D & S1B). The correlation analysis in GBM dataset was also shown in Figure S1. Additionally, we further assessed the histological expressions of LDHA, MCT1, CD68 and CD163 by tissue immunofluorescence staining. In GBM tissue slides, we observed elevated expressions of LDHA and MCT1, along with conspicuous infiltrations of CD68^+^SOX2^−^ TAM and CD163^+^SOX2^−^ M2-TAM. In contrast, very weak or even no signals were observed in Peri-NT and LGG slides (Figs. 1E & S1E).

In summary, lactate metabolism (including production and efflux) was heightened in glioma, particularly in GBM. High levels of lactate metabolism indicated unfavorable survival outcomes for glioma patients. Furthermore, the level of lactate metabolism was positively correlated with M2-subtype TAM infiltration.

### Tumor cells-derived lactate induced tumor-associated macrophages towards M2-polarization in glioma

**Fig. 2:**
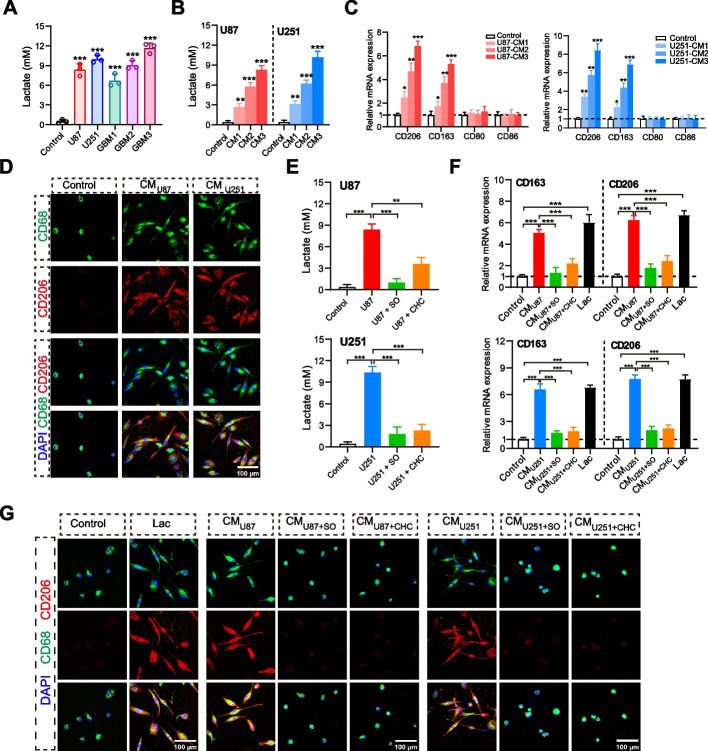
Tumor cells-derived lactate induced tumor-associated macrophages towards M2-polarization in glioma. **A** Lactate concentrations in CMs from glioma cell lines U87, U251, and three cases primary GBM cells. **B** Lactate concentration gradient in CMs from U87 and U251 cells. **C** Quantification of CD206, CD163, CD80 and CD86 mRNA expression in THP1- differentiated macrophages treated with CMs from U87 and U251 cells for 48 h. **D** Representative pictures for immunofluorescence staining for CD68, CD206 and DAPI of macrophages following stimulation with CMs from U87 or U251 cells for 48 h. **E** Lactate concentrations in CMs from U87 or U251 cells treated with SO or CHC. **F** Quantification of CD206 and CD163 mRNA expression in macrophages stimulated with CMs from U87 and U251 cells treated with or without SO and CHC. **G** Representative pictures for immunofluorescence staining for CD68, CD206 and DAPI of macrophages following stimulation with CMs from U87 or U251 treated with or without SO and CHC. Data are presented as the Mean ± SEM. *p < 0.05, **p < 0.01, and ***p < 0.001

To investigate the relationship between lactate derived from tumor cells and tumor-associated macrophages, we collected the conditioned medium (CM) from glioma cells, and employed it to stimulate THP1-differentiated M0-macrophage. It has been documented that PMA treatment could induce differentiation of human monocyte line THP-1 into M0-macrophage [32, 33]. Firstly, we detected lactate concentrations in the cultural supernatants from various glioma cell lines U87, U251, and three cases primary GBM cells. After cultured in serum-free medium for 48 h, the lactate concentrations in the supernatants from glioma cells were detected as range from 6.70 mmol/L to 11.67 mmol/L, in stark contrast to the fresh medium (Fig. 2A). By utilizing the concentration gradient of tumor cells, we established lactate concentration gradient of CMs from U87 (2.68, 5.77 and 8.27 mmol/L), U251 (3.13, 6.23 and 10.17 mmol/L), and GBM3 cells (2.90, 6.033 and 10.53 mmol/L) which were subsequently used to treat M0-macrophage (Figs. 2B & S2A). As illustrated in Figs. 2C & S2B, the treatment with CMs from glioma cells resulted in elevated expressions of M2-macrophage markers CD206 and CD163, while showing no significant alterations in the M1-macrophage markers CD80 and CD86.

Moreover, previous studies have demonstrated that alterations in cell morphology offer a valuable means for evaluating the polarized phenotype of macrophages. Typically, M2-type TAMs exhibit a stretched and elongated morphology [18, 34]. In line with these observations, we noted the stretched and elongated morphology of macrophages after exposure to CMs from glioma cells (Figs. 2D & S2C). Alongside these morphological shifts, CMs from glioma cells also enhanced the expression of surface marker CD206 (Fig. 2D), instead of CD86 (Fig. S2D), indicating a shift towards M2-like polarization, evidenced by immunofluorescent staining.

To underscore the pivotal role of lactate in mediating the polarized effects of macrophages, we treated glioma cells with an LDHA inhibitor, sodium oxamate (SO), to inhibit lactate production, and an MCT inhibitor, α-cyano-4-hydroxycinnamate (CHC), to reduce lactate efflux, respectively [15, 21, 24, 35]. These treatments significantly reduced lactate levels in the supernatants of glioma cells (Figs. 2E & S2E). Subsequently, we stimulated M0-macrophages with CMs from glioma cells that had been subjected to treatment with or without SO and CHC. The reduced lactate levels in CMs led to the absence of upregulated M2-macrophage markers CD206 and CD163 (Figs. 2F & S2F-G). Additionally, as a positive control, we treated M0-macrophages with lactate (10 mM) alone, which also substantially increased the expressions of CD206 and CD163, as determined by qPCR. Similarly, akin to the molecular markers, stimulation with lactate in isolation or CMs from glioma cells induced macrophages to adopt the elongated and stretched morphology, which was not observed when lactate levels in CMs were downregulated using SO or CHC (Figs. 2G & S2H).

Overall, these results demonstrated that the lactate derived from the glioma cells drives TAMs towards an M2-polarized phenotype, thereby remodeling the TME in gliomas.

### Lactate-stimulated M2-TAMs promoted glioma cells proliferation, migration, invasion, and mesenchymal transition

The M2-subtype tumor-associated macrophages (M2-TAMs) have been reported for its role in driving the malignant progression of tumor cells, including proliferation, migration, and invasion. In this study, we sought to investigate the effects of lactate-stimulated M2-macrophages on the malignant behaviors of glioma cells.

**Fig. 3:**
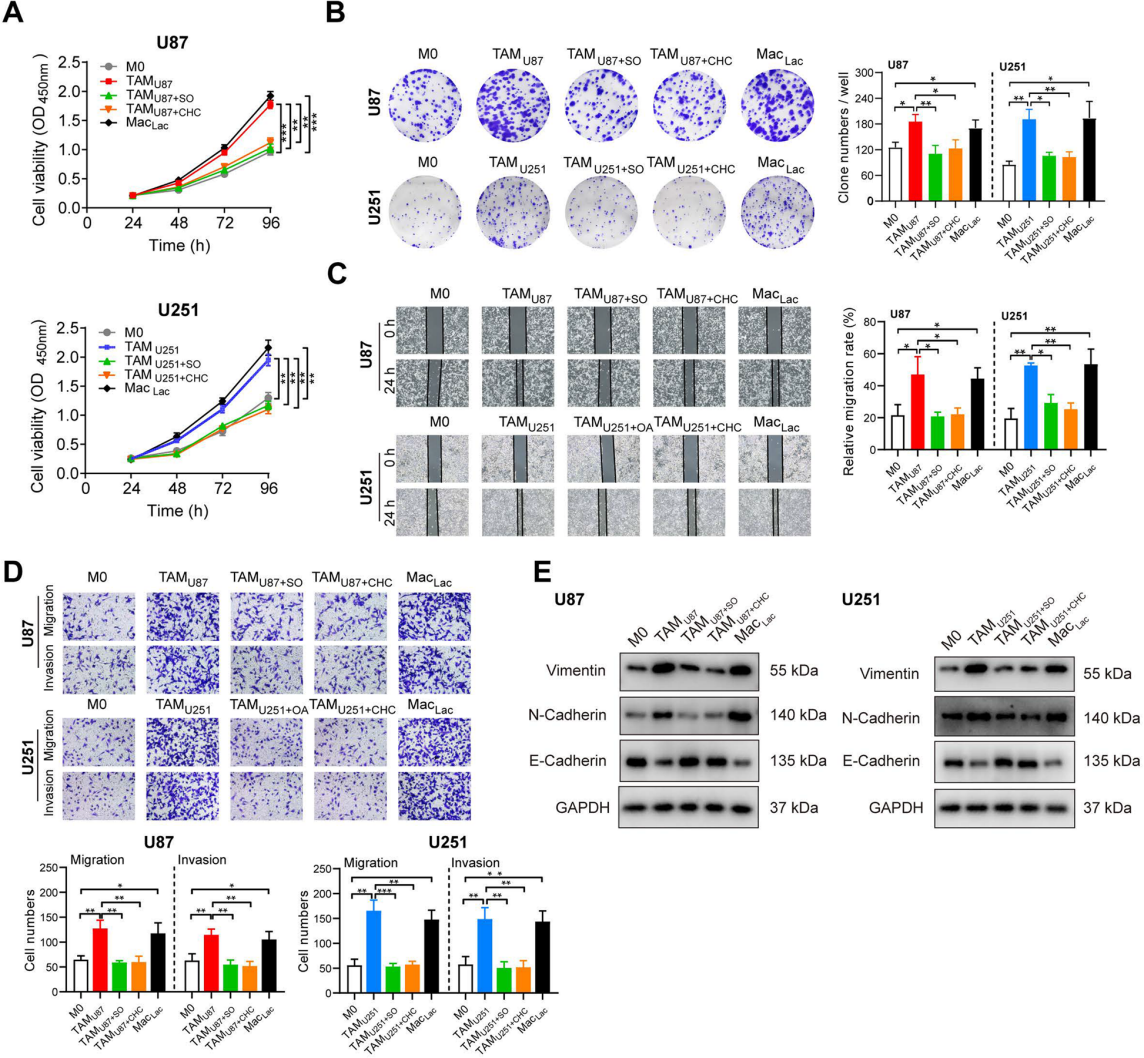
Lactate-stimulated M2-TAMs promoted glioma cells proliferation, migration, invasion, and mesenchymal transition. **A**-**B** Cell proliferation of U87 and U251 cells stimulated with conditioned media (CMs) from various pre-treated macrophages was assessed using the CCK-8 assay (**A**) and the clone formation assay (**B**-**C**). **C**-**D** Cell migration and invasion of U87 and U251 cells stimulated with CMs from various pre-treated macrophages were evaluated through wound healing assays (**C**) and Transwell assays (**D**). **E** Western blot analysis was performed to determine the expression levels of Vimentin, N-cadherin, and E-cadherin proteins in U87 and U251 cells stimulated with CMs from various pre-treated macrophages. Data are presented as the Mean ± SEM. *p < 0.05, **p < 0.01, and ***p < 0.001

Cell proliferation was assessed through both the CCK8 assay and the clone formation assay. We collected conditioned media from various macrophage treatments as described above, to examine their effects on the glioma cells. In comparison with the CMs from M0-macrophages, CMs from the lactate-stimulated M2-macrophages significantly increased the proliferation of glioma cells (Figs. 3A-B & S3A-C). Conversely, cell proliferation declined when lactate levels were reduced by SO and CHC. For migration and invasion, we conducted wound-healing migration and Transwell assays without Matrigel to assess the migratory capacity of glioma cells, and Transwell assays with Matrigel to evaluate invasion. CMs from lactate-treated macrophages notably boosted the motility of glioma cells, evidenced by a reduced scratch area and an increased number of migrating cells (Figs. 3C-D & S3D-G). In contrast, the migration and invasion capabilities of tumor cells were attenuated when the macrophages were stimulated with decline lactate levels. The molecular characteristics of MES subtype consistently indicate a more aggressive tumor state. The PN-to MES transition is recognized as the hallmark of malignant tumor progression, characterized by the upregulation of mesenchymal markers N-cadherin and Vimentin, and the downregulation of epithelial marker E-cadherin [6, 36, 37]. In the final analysis, we detected the expressions of proteins reflecting the malignance at the molecular levels. The expressions of N-cadherin and Vimentin were significantly upregulated by the treatment with lactate-stimulated macrophages, accompanied by the downregulation of E-cadherin. These expressional changes were reversed while the lactate levels were decreased (Figs. 3E & S3H-I).

Above all, these results collectively demonstrated that lactate-stimulated M2-TAMs promote the malignant progression of glioma cells, influencing their proliferation, migration, and invasion, as well as the molecular characteristics of the PN-to-MES transition.

### GPR65 on TAMs serves as the key sensor of lactate-stimulation, fueling glioma cells malignant progression

In the intricate landscape of the tumor microenvironment, the signal transduction triggered by lactate stimulation mainly relays on the lactate receptor HCAR1 (GPR81), and proton-sensing G-protein coupled receptors (GPRs), including GPR4, TDAG8 (GPR65), OGR1 (GPR68), and G2A (GPR132) [38, 39]. Each of these receptors exhibits distinct cellular distribution patterns and can activate various signaling pathways contingent upon specific cell types.

**Fig. 4:**
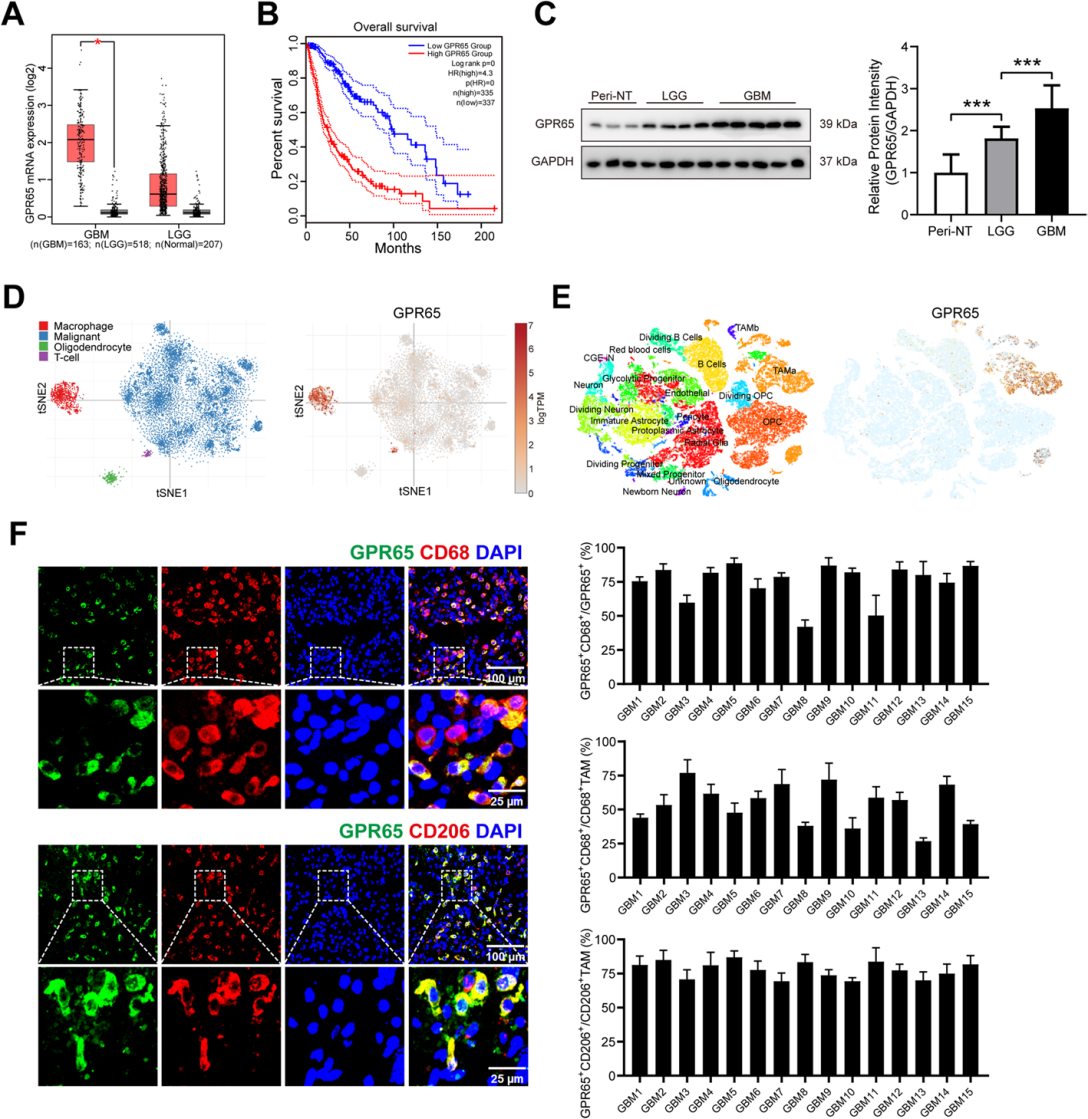
GPR65 was the principal receptor mediating lactate stimulation in TAMs in glioma. **A** Differential mRNA expression levels of GPR65 among *GBM*, *LGG*, and *Normal* groups in TCGA dataset. **B** Kaplan–Meier survival curves for correlation between GPR65 mRNA expression and survival of glioma patients in the TCGA dataset. **C** Western blot analysis and quantification of GPR65 protein expression in clinical samples. **D**-**E** Cellular distribution of GPR65 in glioma using single-cell RNA-seq datasets from UCSC Cell Browser (https://gbm.cells.ucsc.edu) and Single Cell Portal (https://singlecell.broadinstitute.org, GSE131928). **F** Representative pictures and quantification for immunofluorescence staining of clinical tissues for GPR65, and / CD68 or CD206. Data are presented as the Mean ± SEM. *p < 0.05, **p < 0.01, and ***p < 0.001

To investigate which receptor is responsible for the tumor-promoting effects of lactate-stimulation on TAMs in glioma, we analyzed the differential expression and cell distribution of these receptors. Our analysis of glioma datasets downloaded from The Cancer Genome Atlas (TCGA) unveiled significant differences in expression patterns between normal and tumor tissues, specifically an upregulation of GPR65, and a downregulation of GPR68 (Fig. 4A). Conversely, GPR4, GPR81 and GPR132 did not exhibit statistically significant differences (Fig. S4A-D). Additionally, an analysis of cell distribution in glioma using single-cell RNA-seq datasets from UCSC Cell Browser (https://gbm.cells.ucsc.edu) [30, 31] and Single Cell Portal (https://singlecell.broadinstitute.org, GSE131928) [11], revealed that only GPR65 was predominantly expressed on macrophages, while the others showed low expression level or ambiguous cell localizations (Fig. 4D-E & S4E-N). Furthermore, Kaplan–Meier analysis based on TCGA-dataset indicated that high expression of GPR65correlates with poor overall survival among glioma patients (Fig. 4B). This finding was corroborated by the higher expression of GPR65 in GBM samples than in peri-tumorous normal tissues (Fig. 4C). To ascertain the cellular localization of GPR65 in clinical samples, we assessed the co-expression of GPR65, the pan-macrophage marker CD68, and the M2-macrophage marker CD206 in 15 GBM specimens. Immunofluorescence analysis revealed substantial co-localization of GPR65 signals (42%-88%) with the macrophage marker CD68 (Fig. 4F). Furthermore, it was observed that GPR65 was co-expressed in 26%-77% of CD68 + TAMs and 69%-87% of CD206 + TAMs across different samples (Fig. 4F). In summary, these results collectively underscore that among the receptors implicated in lactate signaling, GPR65 is prominently expressed in GBM, with its primary localization on tumor-associated macrophages, especially M2-TAMs.

**Fig. 5:**
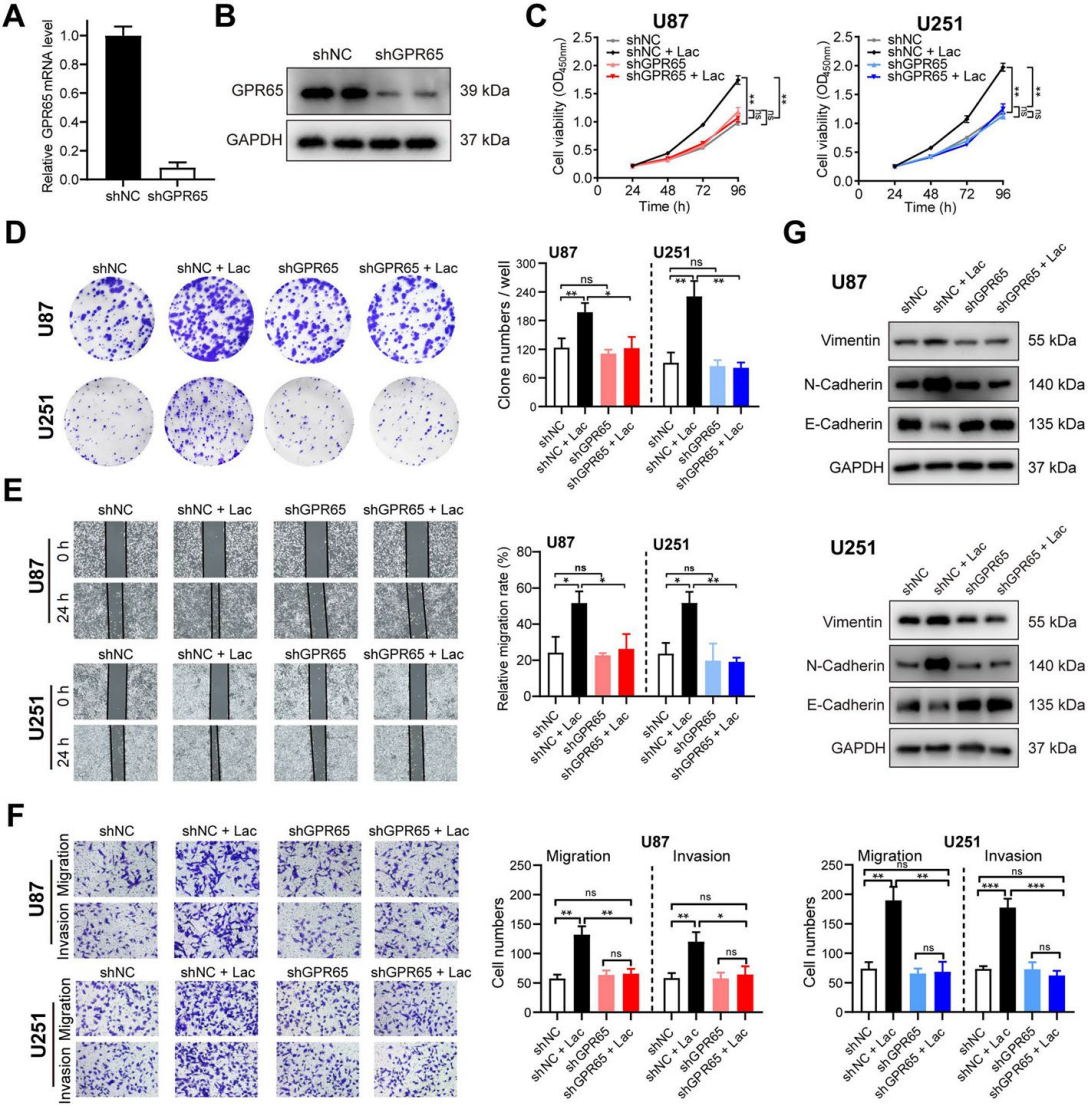
GPR65 on TAMs is essential for lactate-stimulation to promote glioma cells malignant progression. **A** RT-PCR and (**B**) Western blot analysis of GPR65 expression in macrophages transfected with shNC or shGPR65. **C**-**D** CCK8 (**C**) and clone formation assays (**D**) were performed to assess cell proliferation of U87 and U251 cells upon stimulation with CMs from TAMs transfected with shNC or shGPR65 following lactate stimulation. **E**–**F** Wound healing (**E**) and transwell assays (**F**) were conducted to evaluate cell migration and invasion of U87 and U251 cells upon stimulation with CMs from TAMs transfected with shNC or shGPR65 following lactate stimulation. **G** Western blot analysis the protein expression levels of Vimentin, N-cadherin, and E-cadherin in U87 and U251 cells upon stimulation with CMs from TAMs transfected with shNC or shGPR65 following lactate stimulation. Data are presented as the Mean ± SEM. *p < 0.05, **p < 0.01, and ***p < 0.001

To validate whether GPR65 on TAMs indeed functions as the critical sensor for lactate signaling in the TME to drive tumor progression, we transfected TAMs with either shNC (negative control shRNA) or shGPR65 (GPR65-silencing shRNA). The efficacy of transfection was confirmed through qPCR and Western blot analysis (Fig. 5A-B). Subsequently, we collected conditioned media from these transfected TAMs, with or without lactate-stimulation, to treat glioma cells. The results revealed that the proliferative effects of lactate-stimulated shNC-TAMs on glioma cells were attenuated by GPR65 knockdown (Figs. 5C & S5A-C). Furthermore, GPR65 silencing alleviated the enhanced cell migration and invasion induced by lactate-stimulated shNC-TAMs in glioma cells (Figs. 5E-F & S5D-G). Consistent with the cellular behaviors, molecular changes related to mesenchymal transition were also reversed, characterized by decreased expression of Vimentin and N-cadherin, and increased expression of E-cadherin in glioma cells when GPR65 was inhibited (Figs. 5G & S5H-I). Together, these data collectively demonstrated that the tumor-promoting effects of TAMs were counteracted by GPR65-silence on TAMs.

In conclusion, these findings collectively demonstrate that GPR65 on TAMs serves as the key sensor for lactate signaling in the tumor microenvironment, driving the malignant progression and mesenchymal transition of glioma cells.

### GPR65 on TAMs promoted glioma cells malignant progression via HMGB1 secretion

**Fig. 6:**
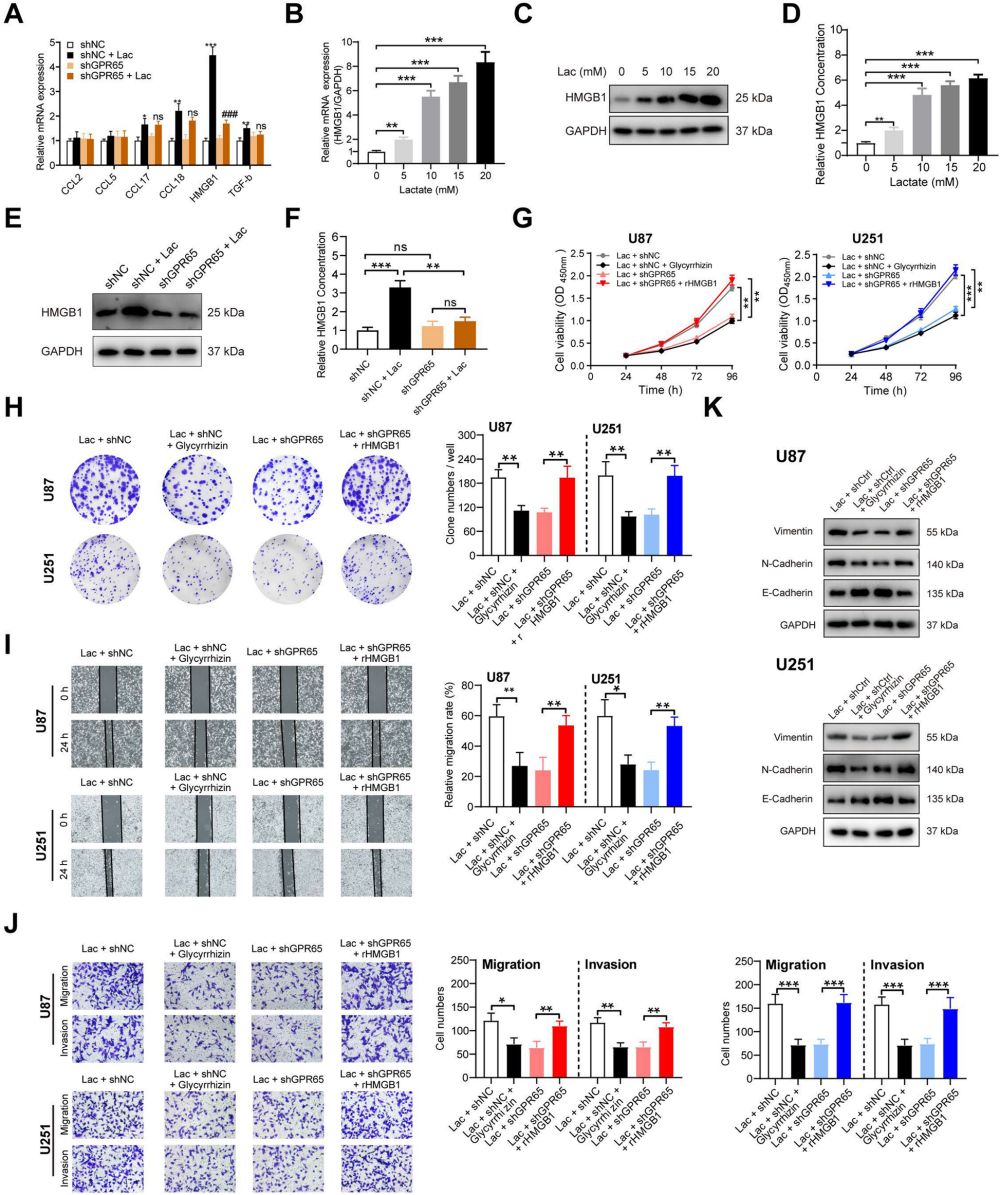
Lactate-stimulation GPR65 on TAMs promoted glioma cells malignant progression via secreting HMGB1. **A** RT-PCR analysis of various potential TAM-derived chemokines and cytokines in PMA-treated THP1 cells transfected with shNC or shGPR65, with or without lactate stimulation. **B** RT-PCR and (**C**) Western blot analysis of HMGB1 expression in macrophages under lactate concentration gradient stimulations. **D** HMGB1 concentrations in CMs from macrophages under lactate concentration gradient stimulations. **E** Western blot analysis of HMGB1 expression in macrophages transfected with shNC or shGPR65, with or without lactate stimulation. **F** HMGB1 concentrations in CMs from macrophages transfected with shNC or shGPR65, with or without lactate stimulation. **G**-**H** CCK8 (**G**) and clone formation assays (**H**) were performed to assess cell proliferation of U87 and U251 cells stimulated with CMs from various pretreated macrophages with HMGB1 inhibitor or rHMGB1. **I-J** Wound healing (**I**) and transwell assays (**J**) were conducted to evaluate cell migration and invasion of U87 and U251 cells stimulated with CMs from various pretreated macrophages with HMGB1 inhibitor or rHMGB1. **K** Western blot analysis of Vimentin, N-cadherin, and E-cadherin protein expression levels of U87 and U251 cells stimulated with CMs from various pretreated macrophages with HMGB1 inhibitor or rHMGB1. Data is presented as the Mean ± SEM. *p < 0.05, **p < 0.01, and ***p < 0.001 vs. shNC group. ^ns^*p* > 0.05, and ^###^*p* < 0.001 vs. shGPR65 group

To gain deeper insights into the mechanism of lactate-induced signaling through GPR65 on TAMs and its role in enhancing the malignant progression of glioma, we analyzed six potential cytokines and chemokines (CCL2, CCL5, CCL17, CCL18, HMGB1, and TGFB1) derived from TAMs, which were previously reported to be associated with lactate levels or pro-tumorigenic activities [14, 15, 18, 33, 40]. We found that the levels of HMGB1 was obviously elevated under lactate stimulation, which were also dropped down when GPR65 on TAMs was silenced (Fig. 6A). To substantiate this observation, we further found that the expressions of HMGB1 were increased along with the lactate concentration gradient at both mRNA and protein levels (Fig. 6B-C). Furthermore, the concentrations of HMGB1 in macrophage supernatants increased in response to lactate stimulation (Fig. 6D). However, the elevation of HMGB1 was inhibited by GPR65-silencing, as confirmed by western blot and ELISA analysis (Fig. 6E-F). Based on these findings, HMGB1 emerged as a potential candidate for validating the pro-tumor effects of lactate-GPR65 signals on TAMs in glioma.

To elucidate the pivotal role of HMGB1 in CMs derived from lactate-stimulated TAMs, we introduced recombinant HMGB1 (rHMGB1) as a supplement into CMs from shGPR65 TAMs to stimulate tumor cells. Concurrently, we employed a selective inhibitor, glycyrrhizin, to reduce HMGB1 levels in CMs from shNC-TAMs. Consequently, the inhibition of HMGB1 levels in CM from shNC TAMs using glycyrrhizin led to a notable reduction of tumor cell proliferation, while the exogenous addition of rHMGB1 rescued the enhanced growth of glioma cells in GPR65-silenced TAMs (Figs. 6G-H & S6A-C). Similarly, rHMGB1 promoted the migration and invasion of glioma cells, while treatment with glycyrrhizin alleviated these processes (Figs. 6H-I & S6D-G). The tendencies of mesenchymal transition induced by rHMGB1 was also mitigated by glycyrrhizin (Figs. 6K & S6H-I). Summarize all, the tumor-promoting effects of lactate stimulated TAMs were mediated by GPR65 through the secretion of HMGB1.

### GPR65 on TAMs activated by lactate stimulates HMGB1 secretion via the cAMP/PKA/CREB signaling pathway

GPR65, as a proton-sensing G-protein coupled receptors, typically triggers an increase in cAMP upon stimulation, subsequently activating the downstream PKA-CREB (PKA: protein kinase A; CREB: cAMP-response element binding protein) signaling pathway [41, 42]. To investigate the signaling molecules responsible for lactate-mediated HMGB1 secretion, we examined changes in this pathway.

**Fig. 7:**
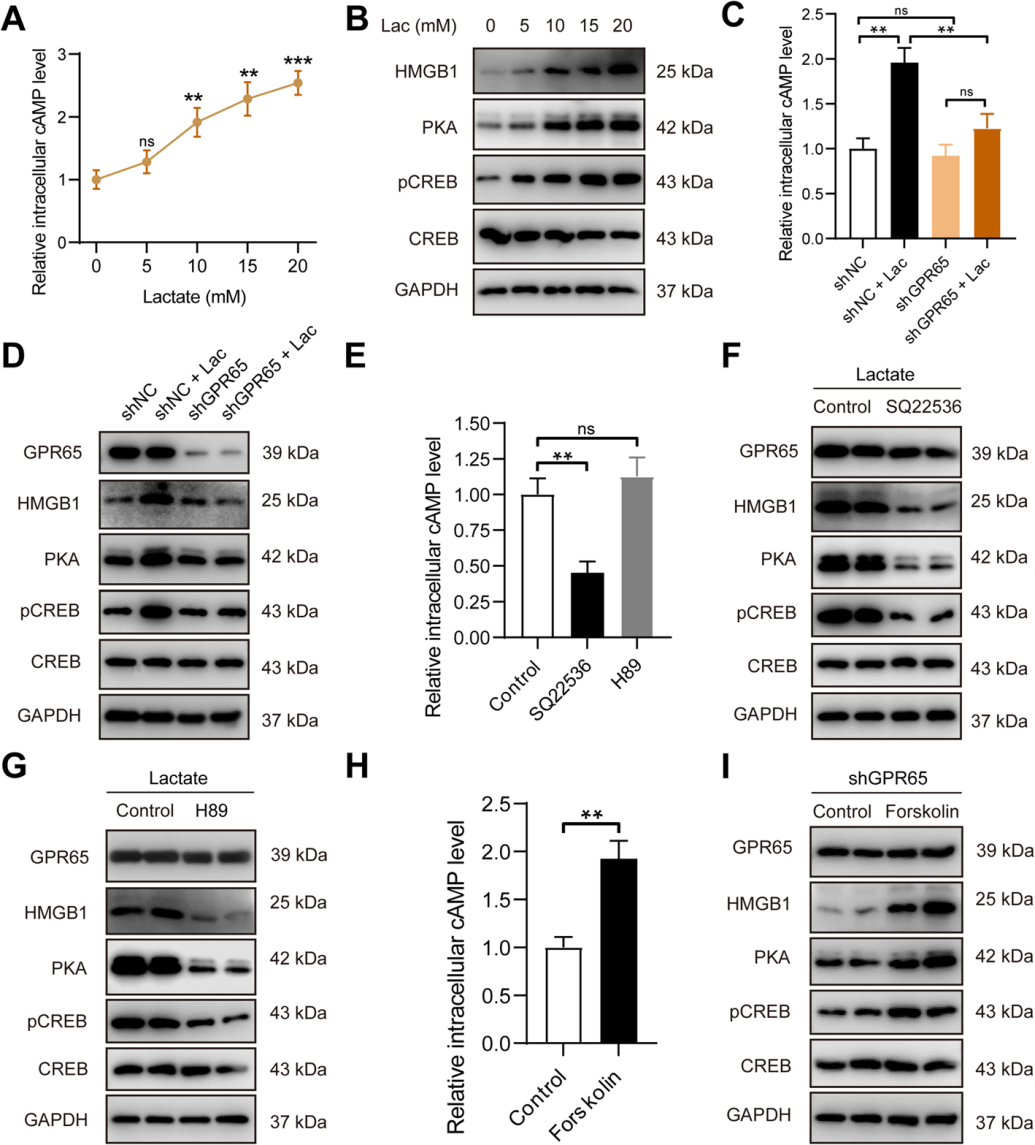
GPR65 on TAMs activated by lactate stimulates HMGB1 secretion via the cAMP/PKA/CREB signaling pathway. **A** Levels of cAMP production in macrophages under lactate concentration gradient stimulations. **B** Western blot analysis of expressions HMGB1, PKA and pCREB in macrophages under lactate concentration gradient stimulations. **C** Levels of cAMP production in macrophages transfected with shNC or shGPR65, with or without lactate stimulation. **D** Western blot analysis of expressions HMGB1, PKA and pCREB in macrophages transfected with shNC or shGPR65, with or without lactate stimulation. **E** Levels of cAMP production in macrophages treated with SQ22536 and H89, with lactate stimulation. **F** Western blot analysis of expressions HMGB1, PKA and pCREB in macrophages treated with SQ22536 with lactate stimulation. **G** Western blot analysis of expressions HMGB1, PKA and pCREB in macrophages treated with H89. **H** Levels of cAMP production in macrophages treated with Forskolin, with lactate stimulation. **I** Western blot analysis of expressions HMGB1, PKA and pCREB in macrophages treated with Forskolin, with lactate stimulation. Data are presented as the Mean ± SEM. *p < 0.05, **p < 0.01, and ***p < 0.001

We observed an increase in cAMP production with rising lactate levels (Fig. 7A). Furthermore, downstream PKA expression increased, enhancing the phosphorylation of CREB (pCREB), a recognized indicator of cAMP-PKA activation (Fig. 7B). However, GPR65 silencing reduced cAMP production and concurrently inhibited PKA and pCREB expression (Fig. 7C-D). The generation of cAMP is mediated by adenylate cyclase (AC), which catalyzes ATP to release a pyrophosphate [43]. To elucidate the roles of cAMP and PKA involving in HMGB1 secretion regulated by GPR65, we employed SQ22536 to reduce cAMP levels by inhibiting AC activity and H89 to inhibit PKA activation (Fig. 7E). Treatment with SQ22536 and H89 respectively resulted in a downregulation of HMGB1 expression, with both PKA and pCREB expressions being suppressed, while cAMP levels were only decreased by SQ22536, not H89 (Fig. 7F-G). This illustrated that PKA functions as a downstream molecule of cAMP. Additionally, an AC activator, Forskolin, was used to re-activate AC in GPR65-silenced cells for further validation. When AC was re-activated in GPR65-silenced TAMs by Forskolin, cAMP levels increased, along with the phosphorylation of CREB increased, and HMGB1 production was upregulated (Fig. 7H-I).

In summary, GPR65 on tumor-associated macrophages responds to lactate stimulation, leading to HMGB1 secretion through the cAMP-PKA-CREB signaling pathway.

### GPR65 knockdown or HMGB1 inhibition mitigates malignant progression of glioma in vivo

**Fig. 8:**
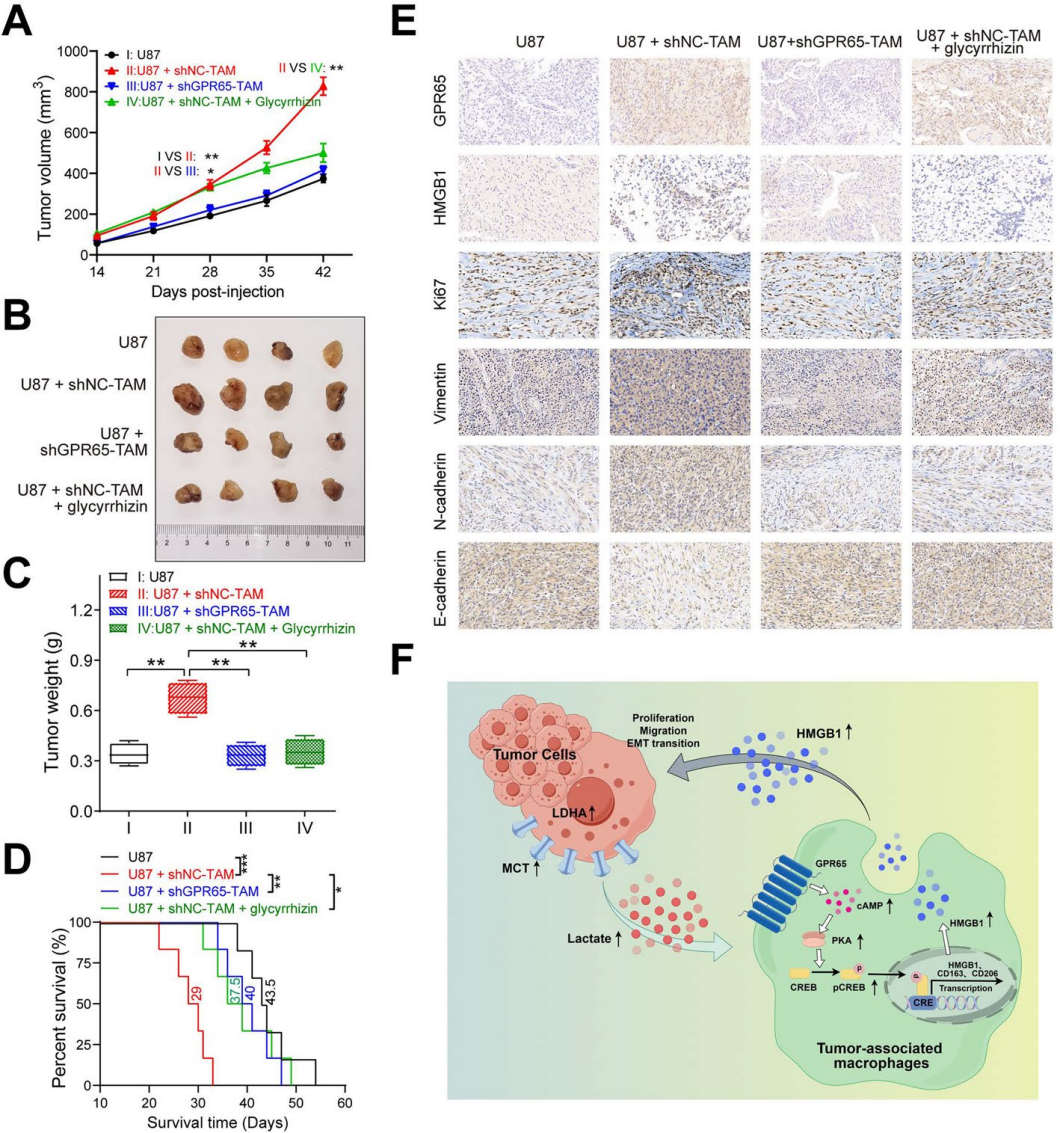
GPR65 knockdown or HMGB1 inhibition mitigates malignant progression of glioma in vivo. **A** In a subcutaneous xenograft tumor model, the tumor volumes were measured and monitored every 7 days from each group of mice. (*n* = 4/group). **B** The pictures of corresponding subcutaneous xenograft tumors dissected from each group mice at 42 days post-injection. **C** Tumor weights from each group at 42 days post-injection. **D** Kaplan–Meier survival curve of each group mice of orthotopic glioma model (*n* = 6/group). **E** Representative images of IHC staining of HMGB1, Ki-67, Vimentin, N-cadherin, and E-cadherin in subcutaneous xenograft tumor. Data are presented as the Mean ± SEM. *p < 0.05, **p < 0.01, and ***p < 0.001

To assess the impact of inhibiting GPR65 or HMGB1 on tumor progression in an in vivo context, we subcutaneously injected U87 cells, with or without tumor-associated macrophages (TAMs), into nude mice to establish subcutaneous xenograft tumors. At 28 days post-injection, it became evident that the group with shNC-TAM (group II) exhibited larger tumor volumes compared to the group injected with tumor cells alone (group I). Conversely, GPR65 knockdown (group III) significantly attenuated tumor growth compared to the shNC-TAM group (group II) (Fig. 8A). Furthermore, at 42 days post-injection, the tumor volume was notably reduced in mice treated with the HMGB1 inhibitor glycyrrhizin (group IV, U87 + shNC + glycyrrhizin) compared to the untreated group (group II, U87 + shNC) (Fig. 8A-B). The corresponding tumor weights at 42 days post-injection followed a similar trend (Fig. 8C).

Additionally, we conducted an orthotopic tumor model to assess the survival of tumor-bearing mice. The results demonstrated that the group with TAMs (group II) exhibited shorter survival compared to the group without TAMs. However, both GPR65 knockdown and HMGB1 inhibition via intraventricular injection of glycyrrhizin extended the survival of the mice (Fig. 8D).

To further elucidate these findings, we conducted immunohistochemistry staining to assess the expression of HMGB1, Ki-67, and mesenchymal transition markers, including Vimentin, N-cadherin, and E-cadherin. TAMs were found to enhance the expressions of HMGB1, Ki-67, Vimentin, and N-cadherin while reducing E-cadherin expression. Conversely, inhibiting GPR65 with shRNA or employing HMGB1 inhibition with glycyrrhizin effectively counteracted these tendencies (Fig. 8E).

In summary, our findings suggest that TAMs promote glioma progression and mesenchymal transition in an in vivo setting. However, the inhibition of GPR65 or HMGB1 alleviates these effects.

## Discussion

Throughout our study, our results unveil the role of lactate in the intricate interplay between tumor cells and tumor-associated macrophages (TAMs), which plays a vital role in promoting malignant progression in glioma. On one hand, tumor cells-derived lactate facilitated macrophages polarization towards M2-subtype. Simultaneously, TAMs respond to lactate stimulation by secreting HMGB1 through the activation of GPR65, further enhancing glioma progression. The mechanism of HMGB1 secretion, driven by GPR65's response to lactate-stimulation, mainly relied on the cAMP-PKA-CREB signaling pathway. Finally, the inhibition of GPR65 or the blockage of HMGB1 effectively alleviated the tumor-promoting effects of lactate-stimulated TAMs.

The Warburg effect commonly leads to the metabolic reprogramming of cancer cells, resulting in increased lactate production and acidosis within the tumor microenvironment, including glioma [44]. Lactate, traditionally viewed as a glycolysis by-product, is now recognized as a significant signaling molecule implicated in tumor progression and various other diseases [45]. Recent research has highlighted the regulatory role of lactate in the immunosuppressive tumor microenvironment, especially in functional polarization of tumor-associated macrophages (TAMs), which promotes tumor progression through the secretion of cytokines and chemokines [15, 17, 46]. Oscar et al*.* [19] previously reported that tumor-derived lactate could induce functional-polarization of TAMs, likely M2-subtype. In breast cancer, tumor-derived lactate was reported to induce macrophage towards M2-polarization via ERK/STAT3 pathway [17]. In pituitary adenoma, Zhang [15] reported that CCL17 chemokine secreted from M2 macrophages promotes tumor invasion via the mTOR/Akt473 pathway. Besides these, M2-TAMs altered by lactate could promote T-cell apoptosis via the PD1/PDL1 pathway, which contributes to assist tumor immune escape [47]. In our study, lactate metabolism levels were elevated significantly with the malignancy grade of glioma. Moreover, the high levels lactate derived from GBM cells stimulated TAMs polarized towards M2-subtype, which facilitated tumor progression in glioma. Our findings reinforce these insights, revealing that elevated levels of lactate play a vital role in regulating the crosstalk between cancer cells and functional TAMs.

The functions of extracellular lactate in the tumor microenvironment depend on its sensing by various receptors on the cell membranes of different cell types, triggering intracellular signaling transduction [48]. Proton-sensing G-protein coupled receptors (GPRs) plays an important role in sensing the lactate signaling, including GPR4, TDAG8 (GPR65), OGR1 (GPR68), and G2A (GPR132). In breast cancer, Gpr132 on BMDMs was reported to sense lactate and mediate TAMs interplay, promoting tumor metastasis [34]. GPR65 was reported to facilitates lung cancer development via PKA/ERK pathway by serving as an extracellular pH sensor [49]. Moreover, GPR65 in GBM was reported as overexpressed and as a predict factor for poor prognosis [50]. Consistent with this, our study showed that among the proton-sensing GPRs, the expression of GPR65 was increased significantly in glioma compared with normal tissues. More importantly, it was revealed that the expression of GPR65 was mainly on TAMs in glioma through analysis of scRNA-seq data and samples verification. Through subsequent functional experiments, the signaling transduction of GPR65 sensing lactate-stimulation was found it necessary for TAMs to promote glioma progression. Overall, among these receptors for lactate-stimulation, GPR65 selectively overexpressed on TAMs in glioma, revealing its potential role as mediator in regulating functional TAMs for tumor progression.

Increasing evidence revealed the vital role of tumor-associated macrophages (TAMs) in facilitating tumor progression, by secreting cytokines or chemokines, which influence cell proliferation, migration, invasion, matrix remodeling and immunosuppression [46]. In breast cancer, a positive feedback loop was reported between mesenchymal-like cancer cells and macrophages via lactate and CCL18, promoting breast cancer metastasis [18]. CCL2 was also reported require for colorectal cancer progression, which was secreted by Wnt5a^+^ TAMs [33]. In pituitary adenoma, lactate-induced M2-TAMs promote pituitary adenoma invasion via secreting CCL17 [15]. As the most abundant immune cells in GBM, TAMs have been the research hotspot for immunotherapy in GBM. TGFB1 was reported secreting from TAMs to promote GBM progression by regulating stem cells growth [13]. In our study, we found that GPR65^+^TAMs could affect the secretion of HMGB1, thereby facilitating the progression and mesenchymal transition of glioma cells. Corresponding to this, HMGB1 also could promote tumor progression in colorectal cancer by activating EMT, Wnt, and ERK signal pathway [14]. In our study, we found that GPR65 sensing lactate-stimulating promotes the expression and secretion of HMGB1 through cAMP/PKA/CREB pathway. HMGB1, which was previously reported as a kind of DAMPs in regulating inflammation, has also been associated with tumor progression now, gradually attracting researchers’ more attention. Thus, the exact mechanisms of HMGB1 in cancer still need more research.

In summary, in the tumor microenvironment, the crosstalk between cancer cells and immune cells frequently occurs, particularly TAMs. In our study, lactate metabolism was elevated in glioma and its high levels in TME stimulated TAMs towards M2-polarization, thereby promoting glioma progression and mesenchymal transition. Moreover, GPR65 was recognized as the main lactate receptor on TAMs in glioma, and its activation drives glioma progression through HMGB1 secretion via the cAMP/PKA/CREB signaling pathway. Inhibiting GPR65 or targeting HMGB1 showed anti-tumor potential for glioma in models, indicating as attractive targets for glioma. While selective GPR65 inhibitors are still in development, they hold significant promise as future therapeutic options.

## Conclusion

In our study, we elucidated the intricate interplay between TAMs and tumor cells mediated by lactate and HMGB1, which significantly promotes tumor progression in glioma. GPR65, found on TAMs, sensed lactate-stimulation and stimulated HMGB1 secretion via the cAMP/PKA/CREB signaling pathway. Blockage this feedback loop presents an attractive therapeutic strategy for glioma.

### Supplementary Information

**Supplementary Material 1.**

## Data Availability

Supplemental data, including figures and tables, are available online. The data used in this study are available upon request.

## Abbreviations

AC: Adenylate cyclase
cAMP: Cyclic adenosine monophosphate
CGGA: The Chinese Glioma Genome Atlas
CHC: α-Cyano-4-hydroxycinnamate
CL: Classical
CM: Conditioned medium
CREB: Cyclic denosine monophosphate response element-binding protein
FBS: Fetal bovine serum
GPR65: G Protein-Coupled Receptor 65
GBM: Glioblastoma
HMGB1: High mobility group box-1 protein
LDHA: Lactate dehydrogenase A
LGG: Low-grade gliomas
MCT: Monocarboxylate transporter
MES: Mesenchymal
NL: Neural
Peri-NT: Peri-tumorous normal tissues
PKA: Protein kinase A
PN: Proneural
SO: Sodium oxamate
TAM: Tumor-associated macrophage
TCGA: The Cancer Genome Atlas
TME: Tumor microenvironment
