# Supplementary material for: GPR65 sensing tumor-derived lactate induces HMGB1 release from TAM via the cAMP/PKA/CREB pathway to promote glioma progression
Source: J Exp Clin Cancer Res. 2024 Apr 4;43:105. doi: 10.1186/s13046-024-03025-8 (PMC10993467; doi:10.1186/s13046-024-03025-8)
Supplement: Supplementary file 1 — Supplementary Material 1. [file 13046_2024_3025_MOESM1_ESM.docx]

**Supplementary Materials**

**Table S1** The antibodies used in this study are provided:

| **Antibodies For WB** | **Source** | **Identifier** |
| --- | --- | --- |
| Rabbit anti-LDHA antibody | Proteintech | Cat #19987-1-AP |
| Rabbit anti-MCT1 antibody | Proteintech | Cat #20139-1-AP |
| Rabbit anti-GAPDH antibody | Proteintech | Cat #10494-1-AP |
| Rabbit anti-Vimentin antibody | Cell Signaling Technology | Cat #5741 |
| Rabbit anti-N-cadherin antibody | Cell Signaling Technology | Cat #13116 |
| Rabbit anti-E-cadherin antibody | Cell Signaling Technology | Cat #3195 |
| Rabbit anti-GPR65 antibody | Proteintech | Cat #20306-1-AP |
| Rabbit anti-HMGB1 antibody | Proteintech | Cat #10829-1-AP |
| Rabbit anti-PKA antibody | Cell Signaling Technology | Cat #4782 |
| Rabbit anti-CREB antibody | Cell Signaling Technology | Cat #9197 |
| Rabbit anti-pCREB antibody | Cell Signaling Technology | Cat #9198 |
| **Antibodies For IHC and IF** | **Source** | **Identifier** |
| Rabbit anti-LDHA antibody | Proteintech | Cat #19987-1-AP |
| Rabbit anti-MCT1 antibody | Proteintech | Cat #20139-1-AP |
| Rabbit anti-CD68 antibody | Abcam | Cat #ab213363 |
| Rabbit anti-CD163 antibody | Cell Signaling Technology | Cat #93498 |
| Goat anti-SOX2 antibody | R&D Systems | Cat #AF2018 |
| Mouse anti-CD86 antibody | Abcam | Cat #ab220188 |
| Goat anti-CD206 antibody | R&D Systems | Cat #AF2534 |
| Mouse anti-CD68 antibody | Proteintech | Cat #66231-2-Ig |
| Rabbit anti-GPR65 antibody | Invitrogen | Cat #PA5111835 |
| Rabbit anti-HMGB1 antibody | Proteintech | Cat #10829-1-AP |
| Rabbit anti-Ki67 antibody | Abcam | Cat #ab15580 |
| Rabbit anti-Vimentin antibody | Cell Signaling Technology | Cat #5741 |
| Rabbit anti-N-cadherin antibody | Cell Signaling Technology | Cat #13116 |
| Rabbit anti-E-cadherin antibody | Cell Signaling Technology | Cat #3195 |

**Table S2** The primer sequences used are provided:

| **Target genes** | **Primer sequences** |
| --- | --- |
| CD206 | Forward, 5’-AGC CAA CAC CAG CTC CTC AAG A-3’;  Reverse, 5’- CAAAAC GCT CGC GCA TTG TCC A-3’. |
| CD163 | Forward, 5’-CCAGAA GGA ACT TGT AGC CAC AG-3’;  Reverse, 5’-CAG GCACCA AGC GTT TTG AGC TA-3’. |
| CD80 | Forward, 5’-CTC TTG GTGCTG GCT GGT CTT T-3’;  Reverse, 5’- GCC AGT AGA TGCGAG TTT GTG C-3’. |
| CD86 | Forward, 5’-CCA TCAGCT TGT CTG TTT CAT TCC-3’;  Reverse, 5’- GCT GTA ATCCAA GGA ATG TGG TC-3’. |
| GPR65 | Forward, 5’-TTG ACT TGA TGC AGG CAC AG-3’;  Reverse, 5’-CCT GGC AAT TGG CTA AAG G-3’. |
| CCL2 | Forward, 5’-CAT CTC CTA CAC CCC ACG AAG-3’;  Reverse, 5’-GGG TTG GCA CAG AAA CGT C-3’. |
| CCL5 | Forward, 5’-CCA GCA GTC GTC TTT GTC AC-3’;  Reverse, 5’-CTC TGG GTT GGC ACA CAC TT-3’. |
| CCL17 | Forward, 5’-GGACGAAGAAGAGCCACAGT-3’;  Reverse, 5’-GCTCCAGTTCAGACAAGGGG-3’. |
| CCL18 | Forward, 5’-CTC TGC TGC CTC GTC TAT ACC T-3’;  Reverse, 5’-CTT GGT TAG GAG GAT GAC ACC T-3’. |
| HMGB1 | Forward, 5’-TAT GGC AAA AGC GGA CAA GG-3’;  Reverse, 5’-CTT CGC AAC ATC ACC AAT GGA-3’. |
| TGFB | Forward, 5’-AAGGACCTCGGCTGGAAGTGC-3’;  Reverse, 5’-CCGGGTTATGCTGGTTGTA-3’. |


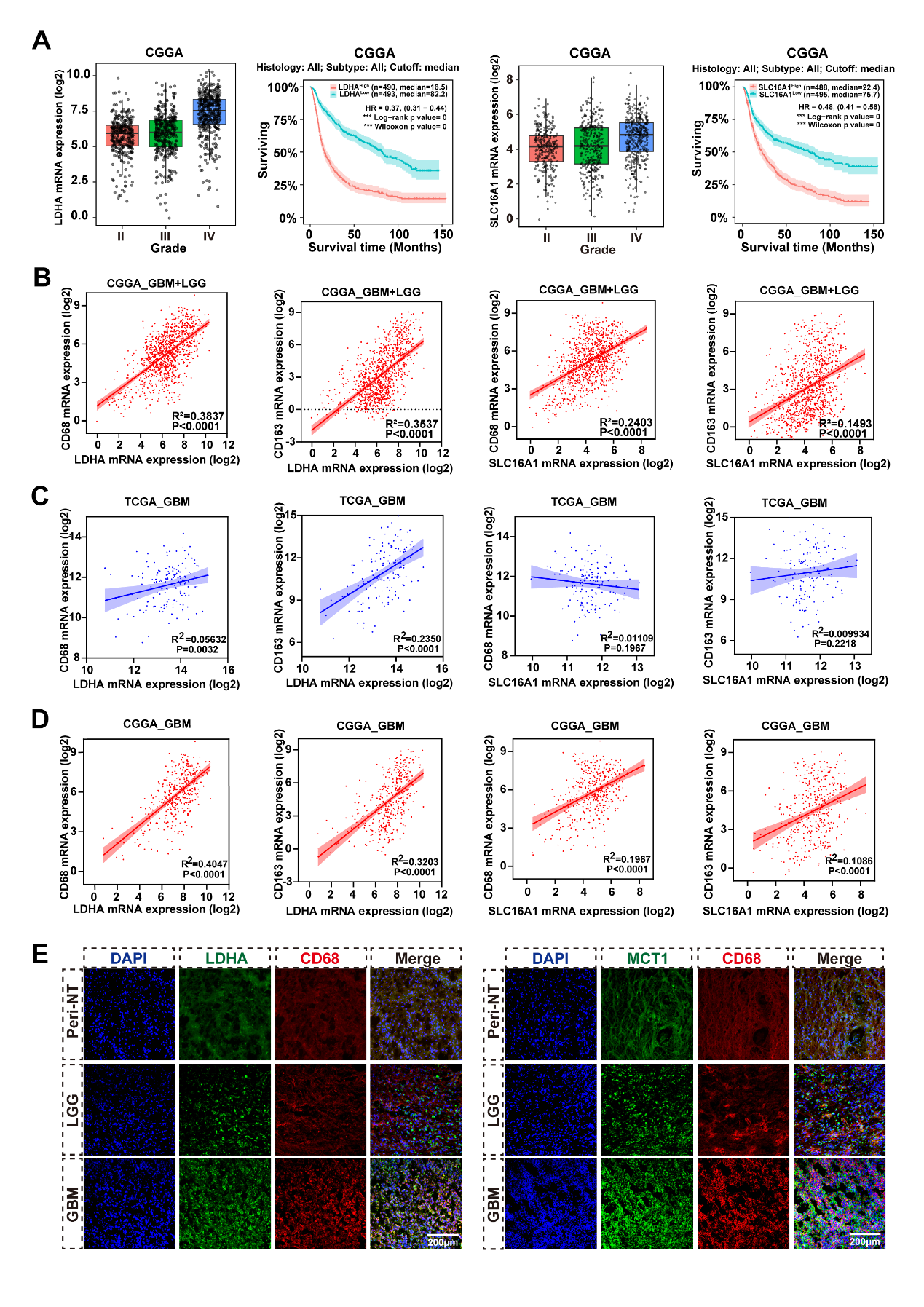
**Figure S1**

**Figure S1 Lactate metabolism was increased in glioma and positively correlated with M2-type TAM infiltration.**

(A) Different mRNA expression levels of LDHA or SLC16A1 among grade of gliomas in CGGA dataset. And Kaplan–Meier survival curves revealed the correlation between LDHA or SLC16A1 mRNA expression and survival of glioma patients in the CGGA datasets. (B) Correlation analysis between the expressions of LDHA or SLC16A1 and CD68 or CD163, respectively, using bulk RNA-seq data from the CGGA dataset. (C-D) Correlation analysis between the expressions of LDHA or SLC16A1 and CD68 or CD163, respectively, using bulk RNA-seq data from the TCGA and CGGA GBM datasets. (E) Representative pictures for co-staining for CD68 and LDHA or CD68 and MCT1 of clinical tissues. Cell nuclei were counterstained with DAPI.


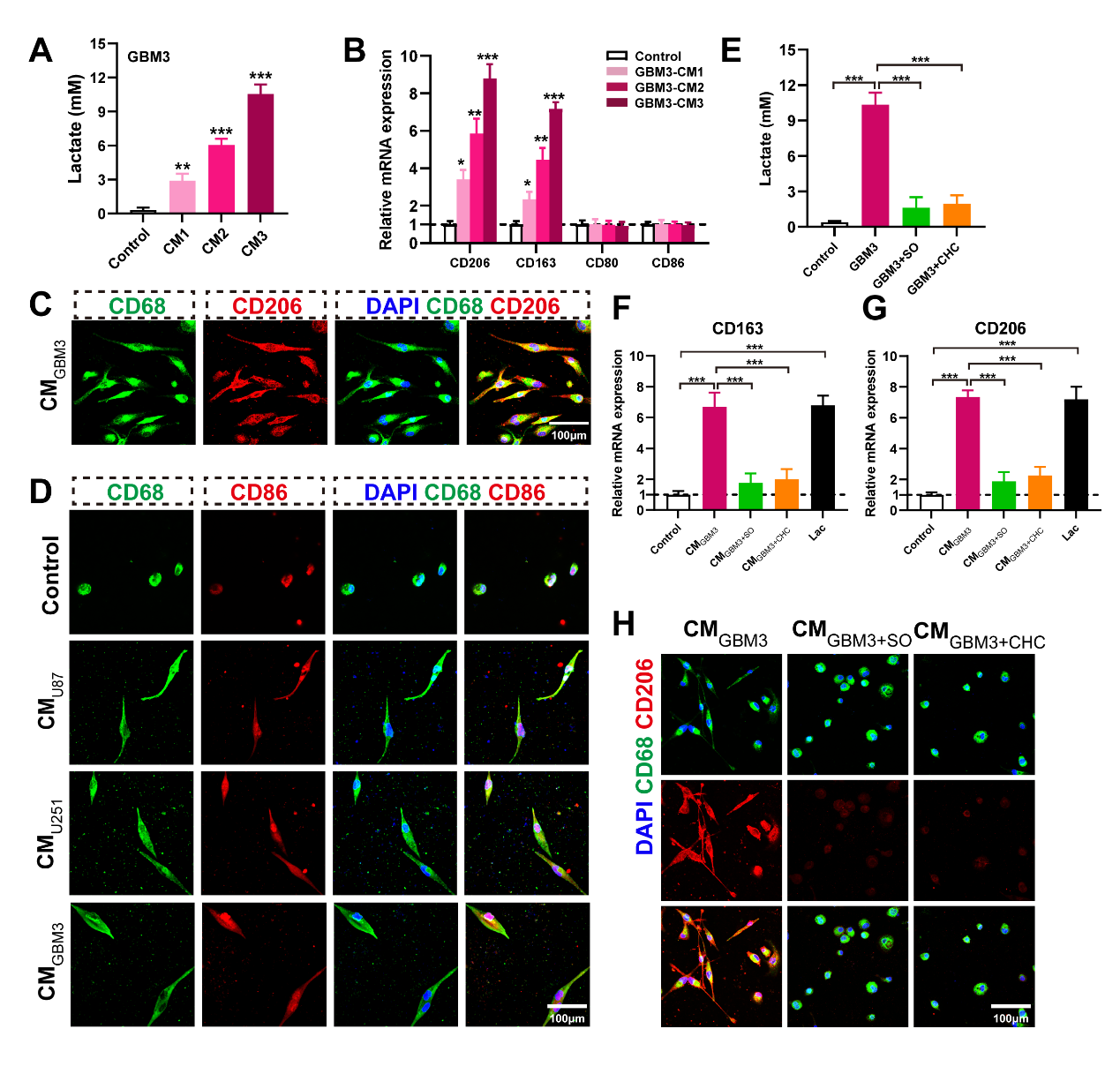
**Figure S2**

**Figure S2 Tumor cells-derived lactate induced tumor-associated macrophages towards M2-polarization in glioma.** (A) Lactate concentration gradient in CMs from GBM3 cells. (B) Quantification of CD206, CD163, CD80 and CD86 mRNA expression in THP1- differentiated macrophages treated with CMs from GBM3 cells for 48 h. (C) Representative pictures for immunofluorescence staining for CD68, CD206 and DAPI of macrophages following stimulation with CMs from GBM3 cells for 48 h. (D) Representative pictures for immunofluorescence staining for CD68, CD86 and DAPI of macrophages following stimulation with CMs from U87, U251, and GBM3 cells for 48 h. (E) Lactate concentrations in CMs from GBM3 cells treated with SO or CHC. (F-G) Quantification of CD206 and CD163 mRNA expression in macrophages stimulated with CMs from GBM3 cells treated with or without SO and CHC. (H) Representative pictures for immunofluorescence staining for CD68, CD206 and DAPI of macrophages following stimulation with CMs from GBM3 treated with or without SO and CHC.


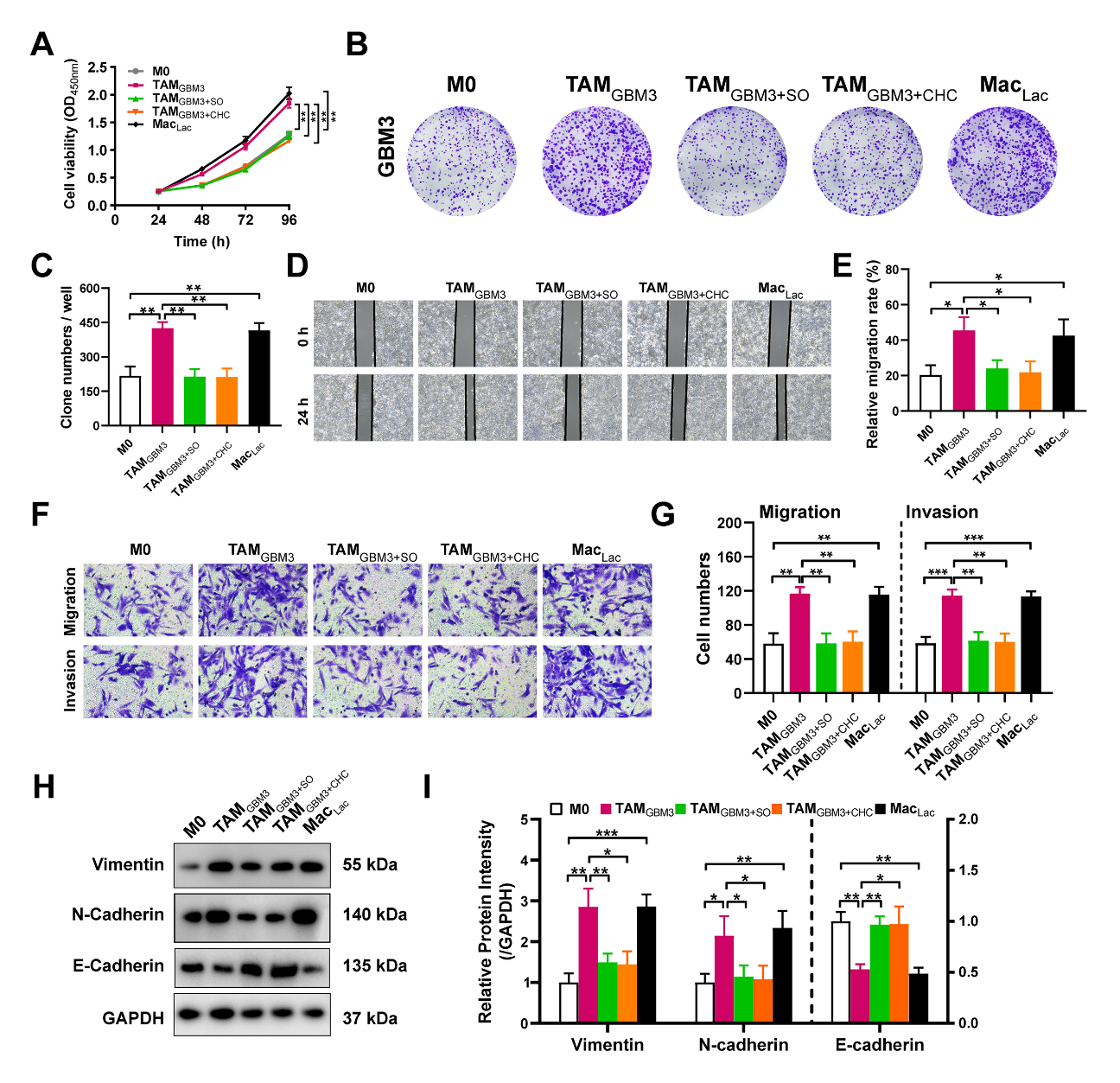
**Figure S3**

**Figure S3 Lactate-stimulated M2-TAMs promoted glioma cells proliferation, migration, invasion, and mesenchymal transition.** (A-C) Cell proliferation of GBM3 cells stimulated with conditioned media (CMs) from various pre-treated macrophages was assessed using the CCK-8 assay (A) and the clone formation assay (B-C). (D-G) Cell migration and invasion of GBM3 cells stimulated with CMs from various pre-treated macrophages were evaluated through wound healing assays (D-E) and Transwell assays (F-G). (H-I) Western blot analysis was performed to determine the expression levels of Vimentin, N-cadherin, and E-cadherin proteins in GBM3 cells stimulated with CMs from various pre-treated macrophages.

**Figure S4**


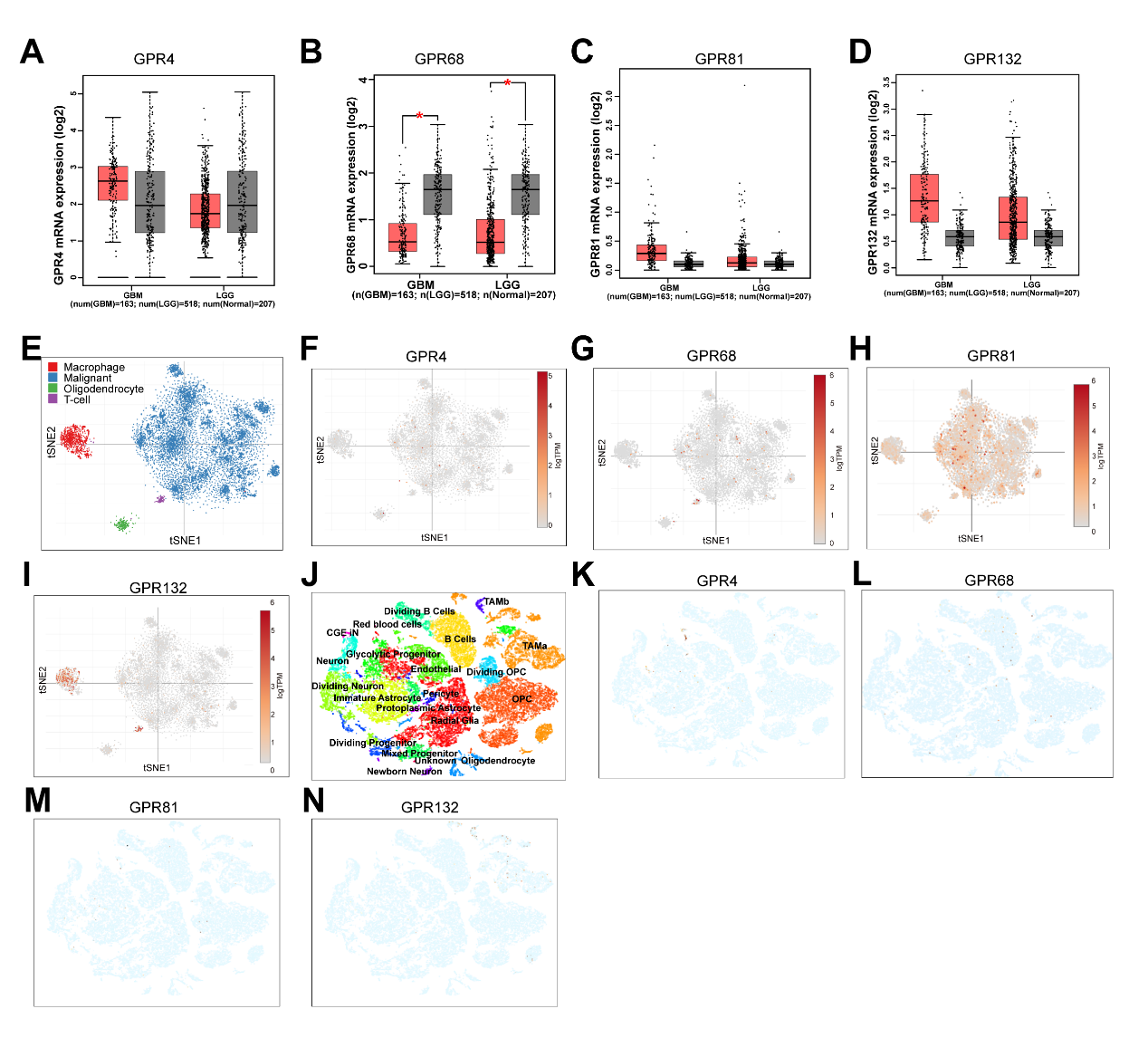


**Figure S4 Differential expression analysis and cellular distribution analysis of potential lactate receptors, such as GPR4, GPR68, GPR81, and GPR132. (A-D)** Differential mRNA expression levels of potential lactate receptors, such as GPR4, GPR68, GPR81, and GPR132 among *GBM*, *LGG*, and *Normal* groups in TCGA dataset. **(E-N)** Cellular distribution of potential lactate receptors, such as GPR4, GPR68, GPR81, and GPR132 in glioma using single-cell RNA-seq datasets from UCSC Cell Browser (https://gbm.cells. ucsc.edu) and Single Cell Portal (https://singlecell.broadinstitute.org, GSE131928).


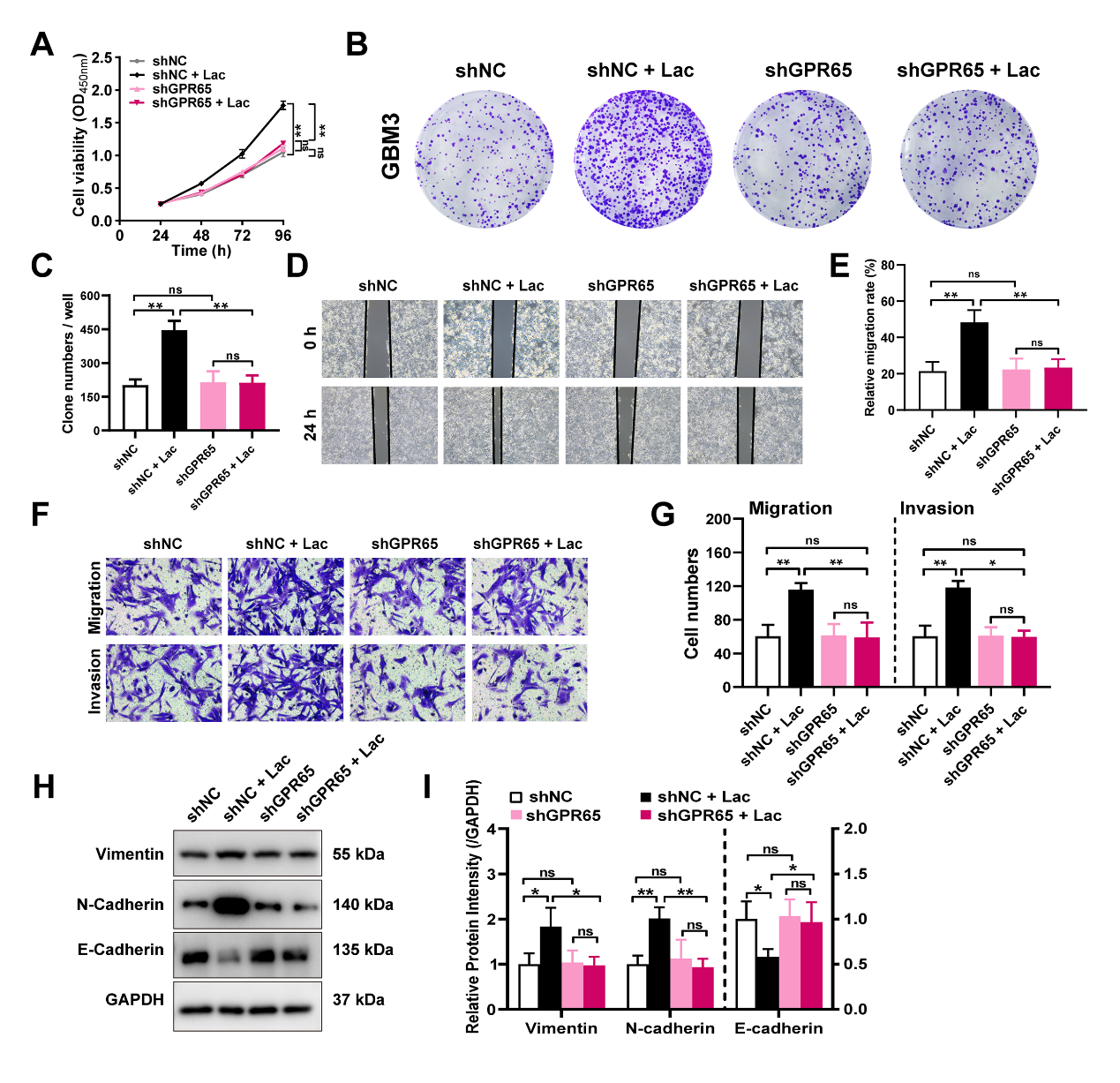
**Figure S5**

**Figure S5 GPR65 on TAMs is essential for lactate-stimulation to promote glioma cells malignant progression.** (A-C) CCK8 (A) and clone formation assays (B-C) were performed to assess cell proliferation of GBM3 cells upon stimulation with CMs from TAMs transfected with shNC or shGPR65 following lactate stimulation. (D-E) Wound healing and transwell assays (F-G) were conducted to evaluate cell migration and invasion of GBM3 cells upon stimulation with CMs from TAMs transfected with shNC or shGPR65 following lactate stimulation. (H-I) Western blot analysis the protein expression levels of Vimentin, N-cadherin, and E-cadherin in GBM3 cells upon stimulation with CMs from TAMs transfected with shNC or shGPR65 following lactate stimulation.


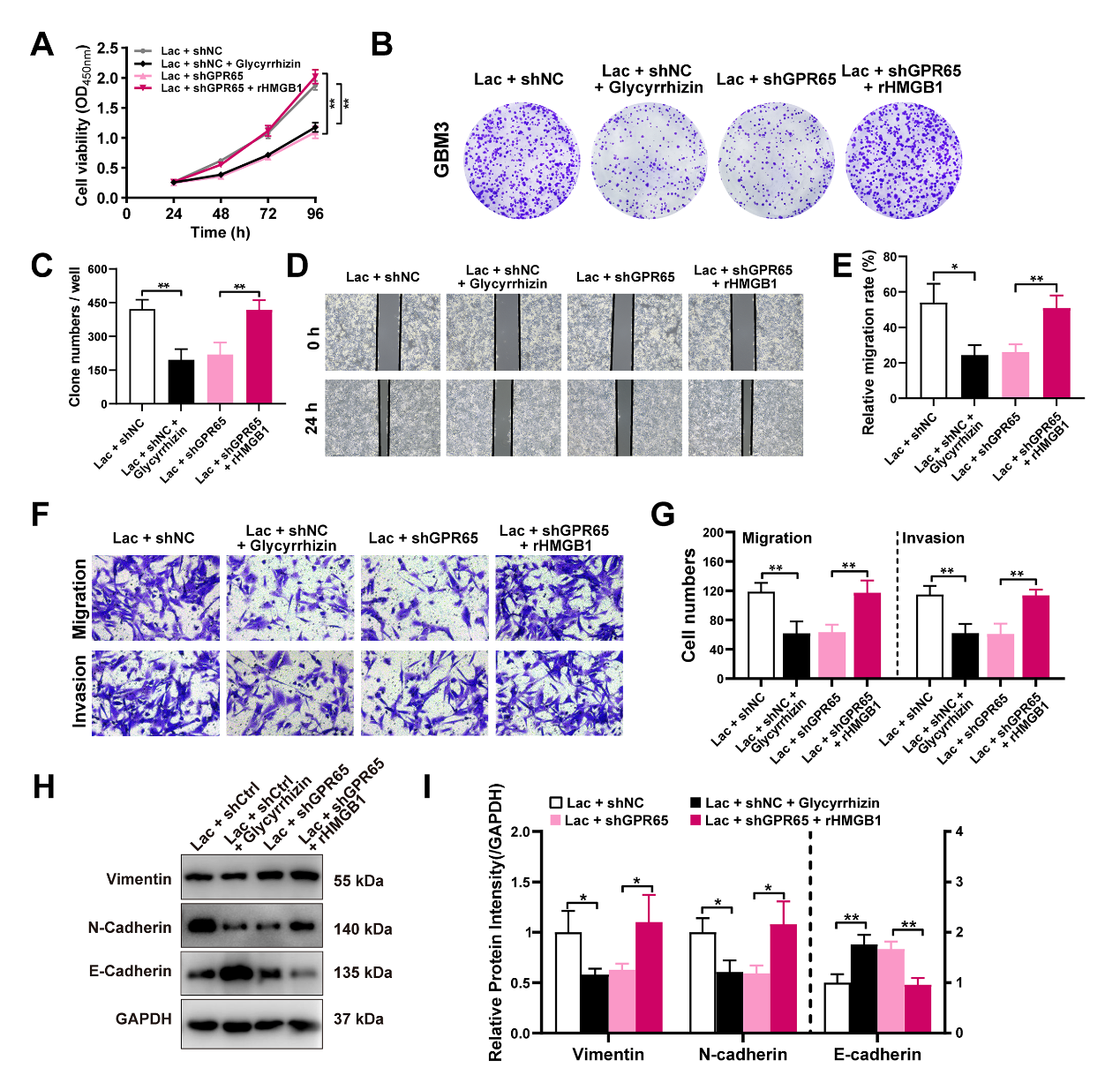
**Figure S6**

**Figure S6 Lactate-stimulation GPR65 on TAMs promoted glioma cells malignant progression via secreting HMGB1.** (A-C) CCK8 (A) and clone formation assays (B-C) were performed to assess cell proliferation of GBM3 cells stimulated with CMs from various pretreated macrophages with HMGB1 inhibitor or rHMGB1. (D-G) Wound healing (D-E) and transwell assays (F-G) were conducted to evaluate cell migration and invasion of GBM3 cells stimulated with CMs from various pretreated macrophages with HMGB1 inhibitor or rHMGB1. (H-I) Western blot analysis of Vimentin, N-cadherin and E-cadherin protein expression levels of GBM3 cells stimulated with CMs from various pretreated macrophages with HMGB1 inhibitor or rHMGB1.
